# African carnivore gut bacterial diversity and composition are associated with sample condition but not storage technique

**DOI:** 10.1186/s42523-026-00553-w

**Published:** 2026-03-24

**Authors:** Laura E. Peirson, Erin A. McKenney, Jessica R. Patterson, James C. Beasley, Stéphanie Périquet-Pearce, Claudine Cloete, Madeline H. Melton, Brennan PetersonWood, Rubén Portas, Ortwin Aschenborn, Diana J. R. Lafferty

**Affiliations:** 1 Wildlife Ecology and Conservation Science Laboratory, Department of Biology, Northern Michigan University, 1401 Presque Isle Ave., Marquette, MI 49855 USA; 2 Conservation Medicine, Smithsonian’s National Zoo and Conservation Biology Institute, Wildlife Health Sciences, Front Royal, VA 22630 USA; 3 Department of Applied Ecology, North Carolina State University, Raleigh, NC 27607 USA; 4 Savannah River Ecology Laboratory, D. B. Warnell School of Forestry and Resources, University of Georgia, Aiken, SC 29802 USA; 5 Ongava Research Centre, Windhoek, Namibia; 6 Panthera, NY USA; 7 Etosha Ecological Institute, Etosha National Park, Windhoek, Namibia; 8 Department of Evolutionary Ecology, Leibniz Institute for Zoo and Wildlife Research of Berlin, Alfred-Kowalke St. 17, 10315 Berlin, Germany

**Keywords:** Animal gut microbiome, African carnivore, Metagenomics, Bioinformatics, Microbiome techniques

## Abstract

Non-invasive fecal sampling is essential for molecular wildlife studies such as gut microbiome (GMB) research, yet field conditions often limit preservation options. To test the effects of preservation methods on the results of GMB community composition, we compared gut bacterial communities in paired fecal samples preserved in stabilization tubes and air-dried in paper bags collected from anesthetized African lions (*Panthera leo*) and spotted hyenas (*Crocuta crocuta*) in Etosha National Park, Namibia. Additional opportunistic samples from the ground around carnivore feeding sites that varied in moisture content were also analyzed. No differences in alpha or beta diversity were detected between preserved and dried samples, although bacterial beta diversity differed between preserved and opportunistic samples, supported by NMDS ordinations and PERMANOVA results. Core bacterial communities remained consistent across opportunistic sample conditions, indicating that host-associated taxa persist despite environmental exposure supporting the use of opportunistic samples for GMB studies in remote arid settings. However, consistent sampling protocols and future field-based desiccation studies remain critical for comparative analyses. These findings highlight that rapid air-drying offers a reliable, low-cost preservation option that maintains core microbiome patterns, expanding the feasibility of GMB research in remote or resource-limited field contexts where refrigeration and preservatives may be unavailable.

**Supplementary Information:**

The online version contains supplementary material available at 10.1186/s42523-026-00553-w.

## Introduction

Exploration of the gut microbiome (GMB) of wildlife has many applications, including species conservation, ecosystem health, and One Health [1]. Research is continuously expanding to include a phylogenetically diverse range of species with varied life history traits and gut morphologies [2]. Amplicon sequencing of the 16 S rRNA gene is a widely used approach in microbiome research but can introduce technical biases during Polymerase Chain Reaction (PCR) amplification [3, 4]. Deep whole-metagenome shotgun sequencing, often exceeding tens of millions of reads per sample, enables comprehensive taxonomic and functional profiling along with directly observed gene profiles, but it is often prohibitively expensive and may generate more data than is necessary for exploratory studies focused on taxonomic composition [3, 5]. As a cost-effective alternative, shallow shotgun metagenomic sequencing performed at 0.5 to 5 million reads per sample avoids PCR amplification and the high data burden and costs associated with deep sequencing [3–5]. Thus, shallow shotgun metagenomic sequencing enables cost-effective exploration of GMB diversity and composition in understudied systems, particularly wildlife.

Host-associated GMBs include the communities of bacteria and eukaryotes (e.g. protists, fungi, and metazoan parasites) that inhabit the digestive tract. Gut bacteria, adapted to the environment within the gastrointestinal tract of their host, are impacted by external environmental factors after defecation [6]. As such, the presence and abundance of gut bacteria can shift in feces post-defecation depending on taxon-specific responses to environmental conditions, and environmental bacteria may colonize and contaminate samples [6]. Thus, immediate preservation of fecal samples best captures GMB composition when collected directly from the host or at the time of defecation [7]. However, collecting fresh fecal samples from wildlife often requires invasive capture techniques, posing health and safety risks to researchers and wildlife. Additionally, capture techniques are difficult to employ in elusive and rare wildlife species [8]. Collecting fecal samples off the ground without inducing stress in target species or risking the safety of humans or wildlife could be a more cost-effective and feasible method of sampling for GMB studies [9]. However, there is a critical need for greater understanding of the extent to which fecal samples are representative of the GMB after exposure to the environment post-defecation, to optimize collection and storage protocols that maintain sample integrity.

Previous studies investigating potential shifts in GMB communities resulting from environmental exposure to fecal samples found varying results. In two African ruminants, springbok (*Antidorcas marsupialis*) and giraffe (*Giraffa camelopardalis*), anaerobic bacteria decreased and both facultatively and obligately aerobic bacteria increased over time since defecation, with faster and more pronounced changes in springbok [6]. In contrast, GMB communities in fecal samples from Rocky Mountain elk (*Cervus canadensis*) collected in Yellowstone National Park remained relatively stable for up to 7 days, with minimal detectable changes in the relative abundance of two genera by day 14 [10]. For spider monkeys (*Ateles geoffroyi*), however, both the preservation method and a 24-hour delay significantly altered GMB diversity and composition [11]. Methodological differences also contributed to variation. For example, in Eurasian cranes (*Grus grus*), invasive (trapped) vs. non-invasive (field-collected) fecal samples differed in richness and composition, with higher richness and composition dispersion in samples from trapped birds, though 60% of genera were shared between methods [9]. Similarly, a time series analysis of cheetah (*Acinonyx jubatus*) fecal subsamples over five days found that alpha diversity and richness were maintained, but relative abundances of some phyla shifted after 24 h, suggesting samples collected within one day of defecation are representative under moist conditions and samples collected after 24 h may introduce compositional biases [12].

Given the variable results in the literature, our goal was to assess the impact of fecal storage techniques (e.g. use of preservation media versus no preservation media) and fecal sample conditions (e.g. moisture content at time of collection) on microbiome community integrity for African carnivore GMB research. This study builds on previous research by evaluating which storage-related effects on microbiome diversity and composition reported in other taxa are most evident in large, free-ranging carnivores under field conditions. We had two main objectives: (1) to compare GMB community composition and diversity between freshly preserved samples collected directly from hosts and placed into a commercially available DNA stabilization buffer containing < 24% ethanol (“Wet” samples; lions *n* = 30, hyenas *n* = 15) and a subset placed in brown paper bags, hung in trees, and dried for 24 h under ambient conditions (“Dry samples; lions n = 16, hyenas = 4); and (2) to compare freshly preserved samples with opportunistically collected samples collected from the environment (“Opportunistic” samples; lions n = 116, hyenas = 41). Opportunistic samples were collected along roads, around waterholes, or near carnivore kill sites, categorized by gross moisture content (e.g. inside–outside soft [IOS], inside soft–outside hard [ISOH], inside–outside hard [IOH], and samples containing only hairs [Hairs]) as a proxy for time since defecation, and were subsequently air-dried under ambient conditions following the same drying protocol used for Dry samples. Additionally, we determined whether the presence or absence of microbial taxa correlated with the sample storage technique or the condition in which samples were collected. We hypothesized that GMB community diversity and composition would not differ between fresh preserved samples and fresh samples that had been dried, and that rapid drying in the semi-arid environment of Etosha National Park in northern Namibia [13] would preserve core GMB taxa regardless of initial sample conditions. We expected opportunistically collected samples to show enrichment of environment-associated taxa (e.g. soil-associated microbes) and shifts in community membership relative to freshly preserved samples.

## Results

### Storage technique

**Fig. 1:**
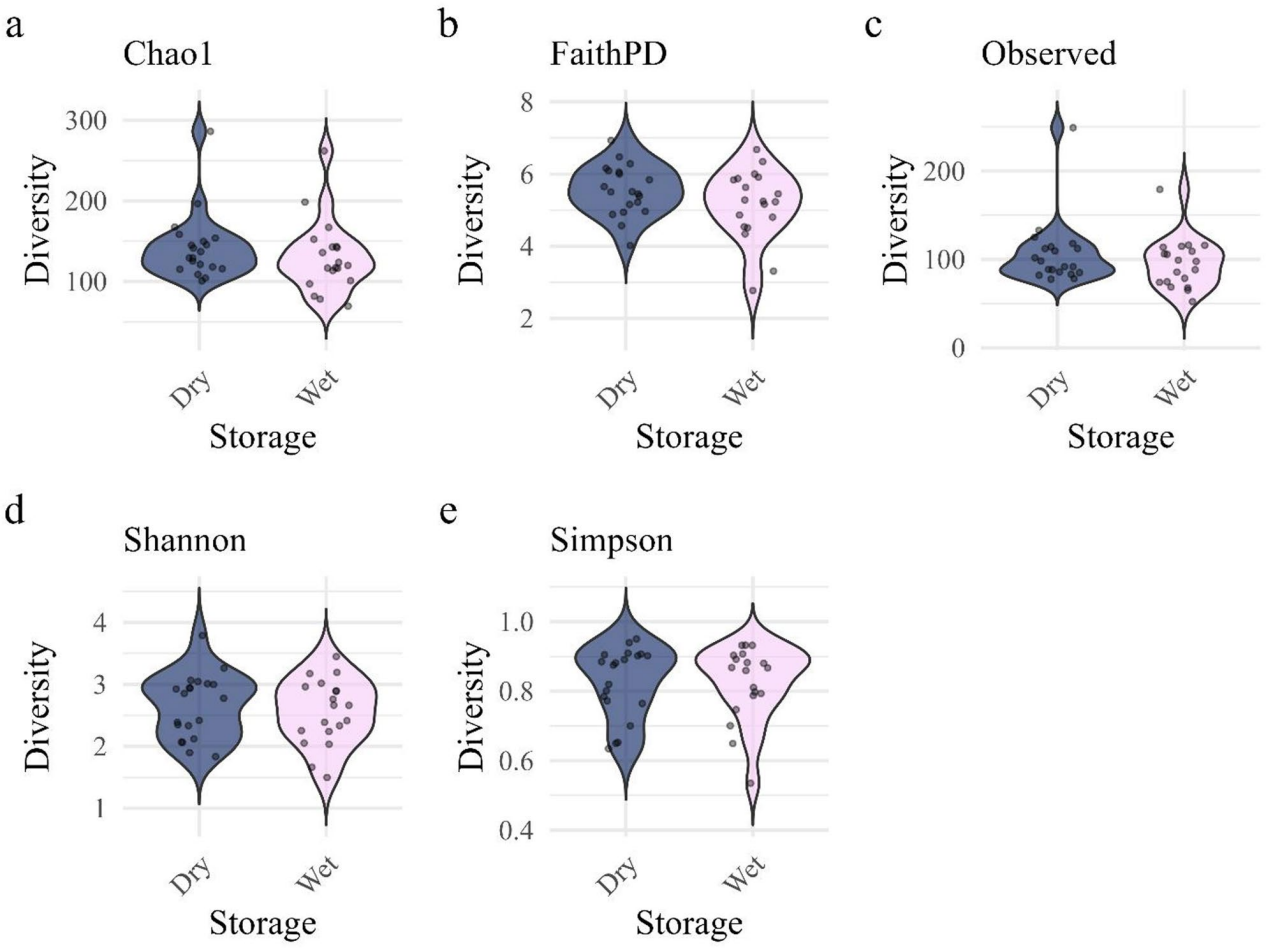
Comparison of alpha diversity metrics between storage techniques across African lion (*Panthera leo*) and spotted hyena (*Crocuta crocuta*) fecal samples. Panels represent: (**a**) Chao1, (**b**) Faith’s Phylogenetic Diversity (FaithPD), (**c**) Observed taxa, (**d**) Shannon diversity, and (**e**) Simpson diversity. Storage technique identification is represented by “Wet” for fresh preserved and “Dry” for fresh, paired dried samples. No significant differences (Paired T-Test, all p-values > 0.050) were observed in alpha diversity metrics between Wet-Dry pairs

**Fig. 2:**
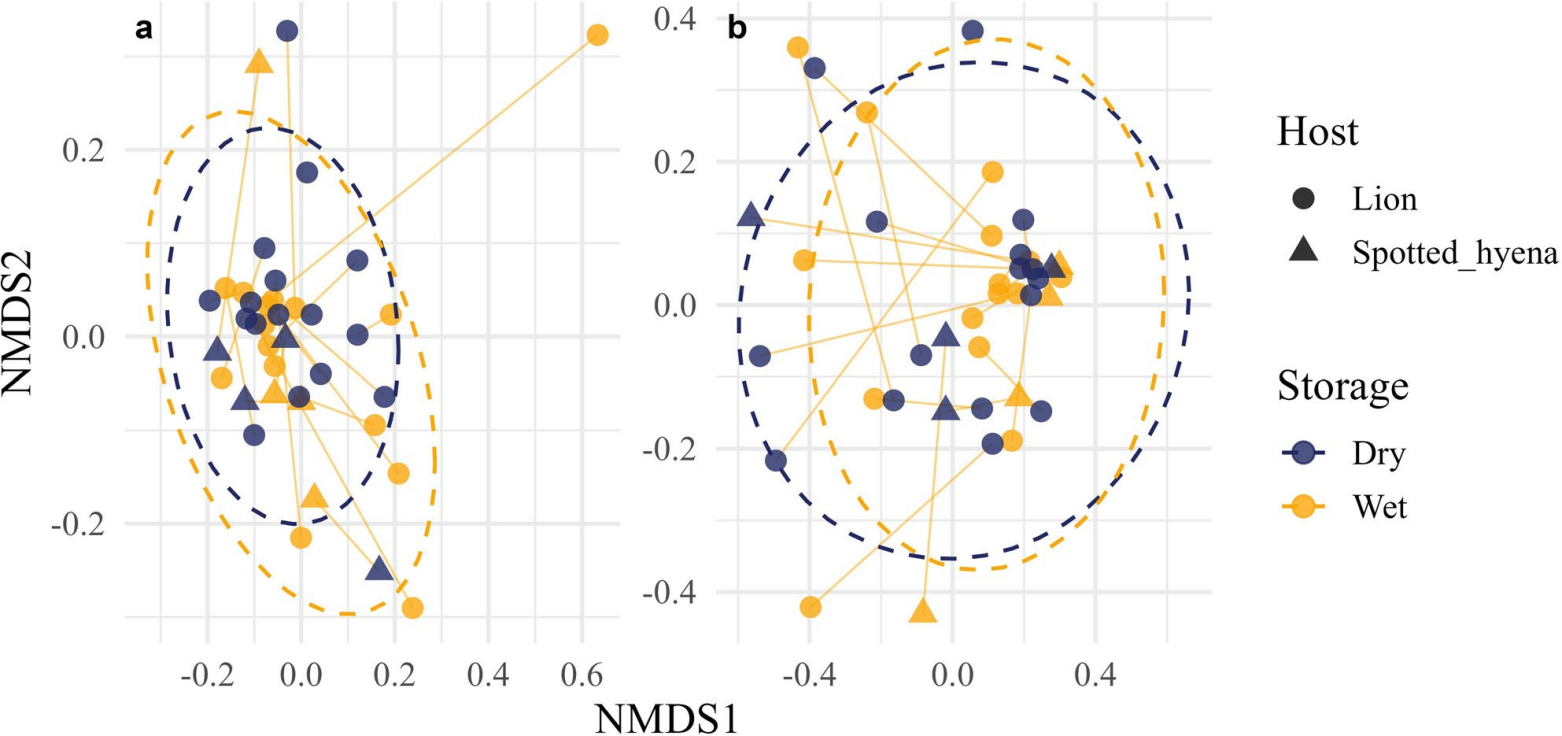
Non-metric multidimensional scaling (NMDS) ordinations of paired fecal samples from African lions (*Panthera leo*) and spotted hyenas (*Crocuta crocuta*) comparing storage techniques. Ordinations are based on (**a**) unweighted UniFrac and (**b**) Bray–Curtis dissimilarity. Points represent individual samples colored by storage technique (Wet vs. Dry) and shaped by host species. Paired samples collected from the same individual are connected by lines, illustrating within-individual similarity between storage treatments. Dashed ellipses represent 95% confidence intervals around group centroids. Storage technique identification is represented by “Wet” for freshly preserved and “Dry” for freshly collected, paired dried samples. No significant differences were observed in UniFrac distances between Wet and Dry samples (*p* > 0.05). NMDS stress values were 0.213 for unweighted UniFrac and 0.071 for weighted UniFrac

No differences in alpha diversity metrics (Observed, Shannon, Chao1, Simpson’s, and Faith’s PD) were observed between storage techniques (Wet vs. Dry) based on paired t-tests (*p* > 0.05, Fig. 1). Similarly, no differences in community composition were detected between Wet and Dry samples using PERMANOVA across all beta diversity metrics, including Bray–Curtis dissimilarities, weighted and unweighted UniFrac distances, and Aitchison distance (all *p* > 0.05; Fig. 2).

**Fig. 3:**
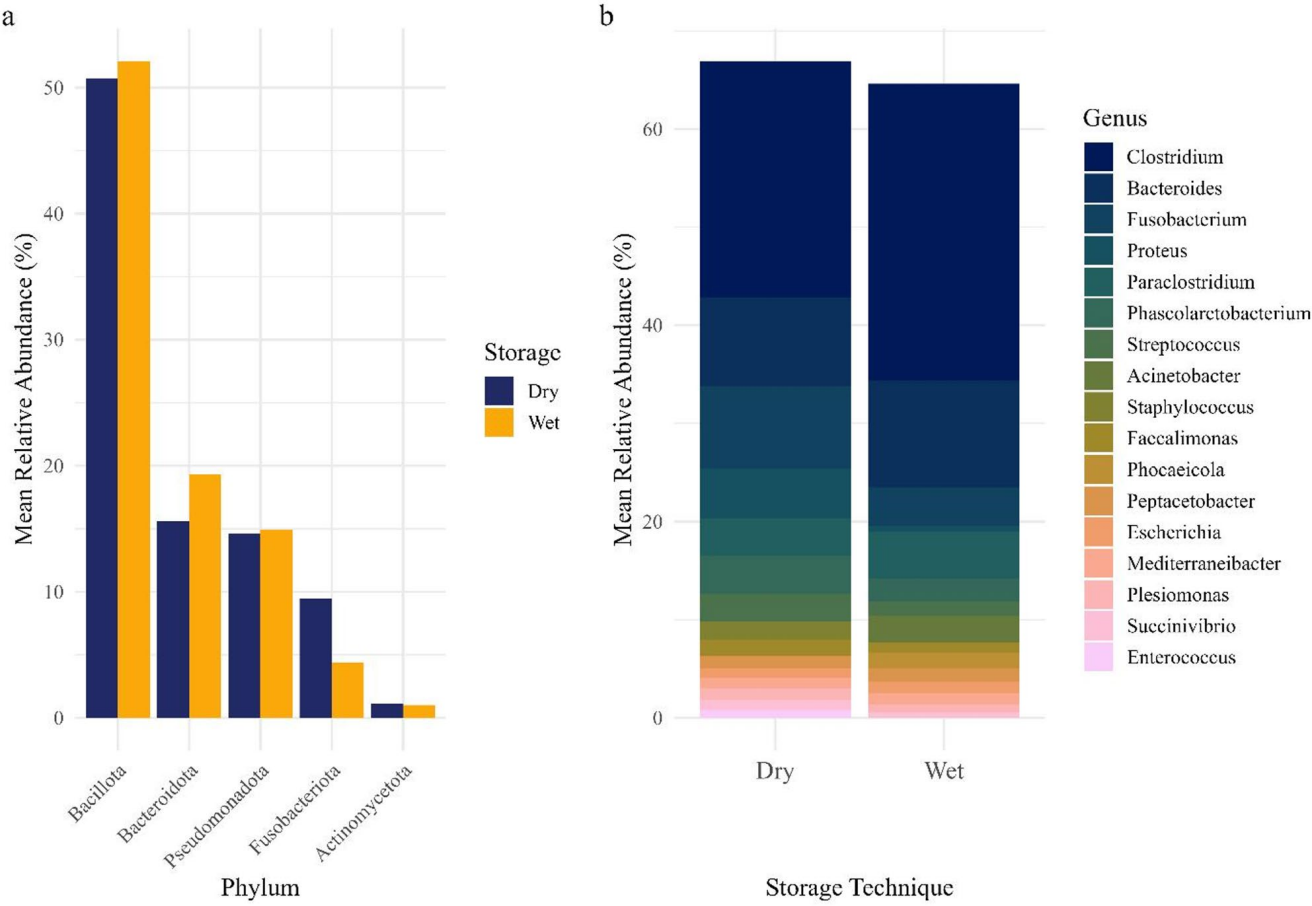
Comparison of (**a**) top phyla and (**b**) top genera by mean relative abundance (> 1% across samples) across African lion (*Panthera leo*) and spotted hyena (*Crocuta crocuta*) samples. Storage technique identification is represented by “Wet” for fresh preserved samples and “Dry” for fresh, paired dried samples. (**a**) The five most abundant phyla by overall mean relative abundance in Wet and Dry samples are shown. (**b**) The fifteen most abundant genera by mean relative abundance in Wet and Dry samples are shown

The most abundant phyla across Wet and Dry samples, based on mean relative abundance, were *Firmicutes* (Wet: 52% / Dry: 51%), *Bacteroidetes* (19% / 16%), *Proteobacteria* (15% / 15%), and *Fusobacteriota* (4% / 9%) (Fig. 3a), consistent with previous findings in carnivores [14]. The most abundant genera, based on mean relative abundance, across Wet and Dry samples were *Clostridium* (Firmicutes, 30%/24%), *Bacteroides* (Bacteroidetes, 11%/9%), and *Fusobacterium* (Fusobacteriota, 4%/8%) (Fig. 3b). Variability in relative abundance among samples is illustrated in Fig. 3.

**Fig. 4:**
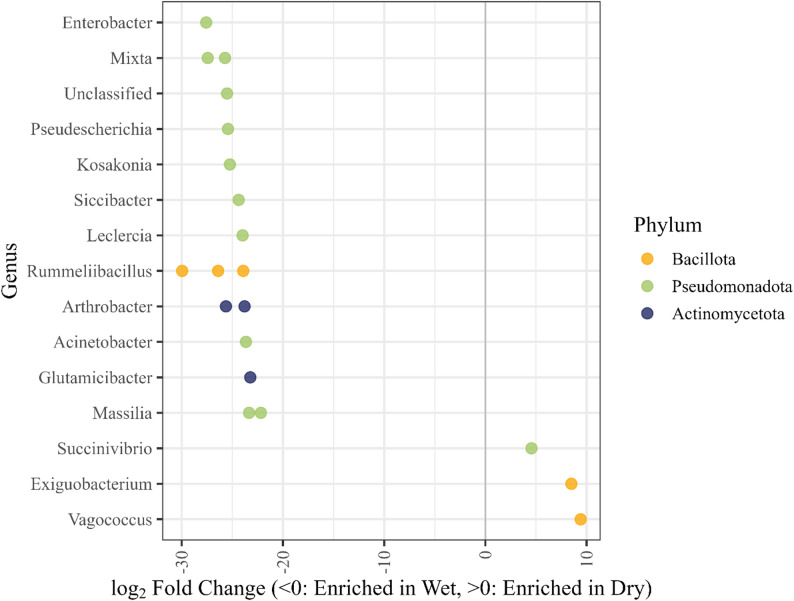
Differentially abundant bacterial genera between Wet and Dry samples across African lions (*Panthera leo*) and spotted hyenas (*Crocuta Crocuta*) based on DESeq2 analysis (α = 0.05). Storage technique identification is represented by “Wet” for fresh preserved and “Dry” for fresh, paired dried samples. Points represent significantly different genera colored by phylum. Each dot represents a bacterial genus identified as significantly differentially abundant between Wet and Dry samples. Genera with positive log₂ fold change values are enriched in Dry samples, while negative values indicate enrichment in Wet samples. *Succinivibrio* was the only genus enriched in Dry samples that exceeded 1% mean relative abundance, while all genera enriched in Wet samples remained below this threshold

DESeq2 analysis identified 17 genera significantly enriched in Wet samples compared to 3 enriched in Dry samples, including *Rummeliibacillus*, *Enterobacter*, and *Mixta*, although none surpassed 1% mean relative abundance (Fig. 4). In contrast, *Succinivibrio*, *Exiguobacterium*, and *Vagococcus* were significantly enriched in Dry samples, with *Succinivibrio* as the only genera to exceed 1% mean relative abundance (Fig. 4). Using ANCOM-BC2, two genera were identified as differentially abundant between storage techniques after FDR correction (q < 0.05). *Succinivibrio* was enriched in Dry samples, whereas *Campylobacter* was enriched in Wet samples. However, neither genus passed the sensitivity analysis for pseudo-count addition, indicating that these differences were not robust to alternative zero-handling assumptions. Accordingly, no taxa were considered robustly differentially abundant between storage treatments. Host-stratified DESeq2 analyses revealed 12 enriched genera in Wet lion samples and 1 enriched genera in Dry lion samples (see Additional file 1), whereas no taxa were significantly differentially abundant among hyena samples after multiple-test correction.

### Sample condition

**Fig. 5:**
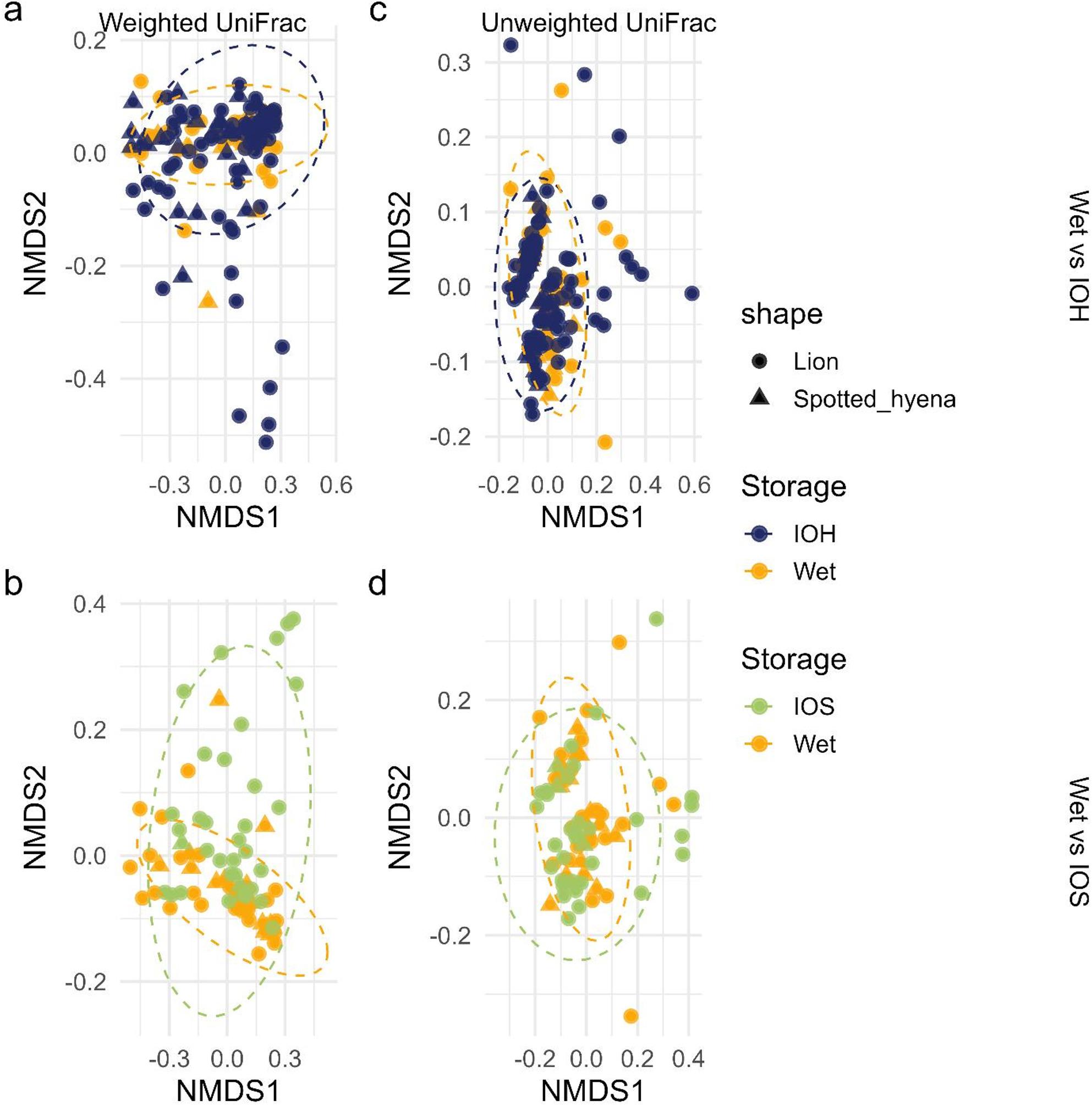
Non-metric multidimensional scaling (NMDS) ordinations based on weighted and unweighted UniFrac distances. Sample condition identification is represented by “Wet” for fresh preserved samples, “IOH” for inside-outside hard samples, and “IOS” for inside-outside soft samples. Points represent individual samples, colored by sample condition and shaped by host species (African lion [*Panthera leo*] and spotted hyena [*Crocuta crocuta*]), with 95% confidence ellipses shown for each group. (**a**) NMDS on weighted Unifrac distances for Wet vs. IOH, stress = 0.084; (**b**) NMDS on unweighted Unifrac distances for Wet vs. IOH, stress = 0.176; (**c**) NMDS on weighted Wet vs. IOS, stress = 0.088; (**d**) NMDS on unweighted Unifrac distances for Wet vs. IOS, stress = 0.176. PERMANOVA revealed significant differences in bacterial community composition between Wet vs. IOS (unweighted UniFrac, *p* = 0.012, R² = 0.002) sample conditions

Significant differences in alpha diversity metrics (Observed, Shannon, Chao1, Simpson, and Faith’s PD) were observed between Wet samples and both IOH and IOS samples (Wilcoxon rank-sum tests, all *p* < 0.050 (except Wet vs. IOS Faith PD, *p* = 0.090), but not between IOH and IOS. Comparisons involving ISOH (lion *n* = 2, hyena *n* = 0) and Hairs (lion *n* = 2, hyena *n* = 2) were excluded due to limited sample sizes and reduced statistical power. Stratified pairwise PERMANOVA (controlling for host species) on Bray–Curtis distances detected differences in GMB community composition between Wet and IOH, and between Wet and IOS (Bonferroni-adjusted *p* = 0.045 and *p* = 0.003, respectively), but not between IOH and IOS. Stratified pairwise PERMANOVA on unweighted UniFrac distances (Bonferroni-adjusted *p* = 0.012) detected significant differences in GMB community composition between Wet and IOS, but not between Wet and IOH or IOH and IOS. Stratified pairwise PERMANOVA on weighted UniFrac distances detected no significant differences among groups (all *p* > 0.050). Stratified pairwise PERMANOVA on Aitchison (CLR–Euclidean) distances detected differences in GMB community composition between Wet and IOH and between Wet and IOS (Bonferroni-adjusted *p* = 0.003 for both comparisons), whereas no differences were detected between IOH and IOS. NMDS ordinations based on weighted and unweighted UniFrac distances (Fig. 5) indicate substantial overlap between Wet and opportunistic samples, with IOS samples exhibiting greater dispersion in ordination space relative to Wet samples.

**Fig. 6:**
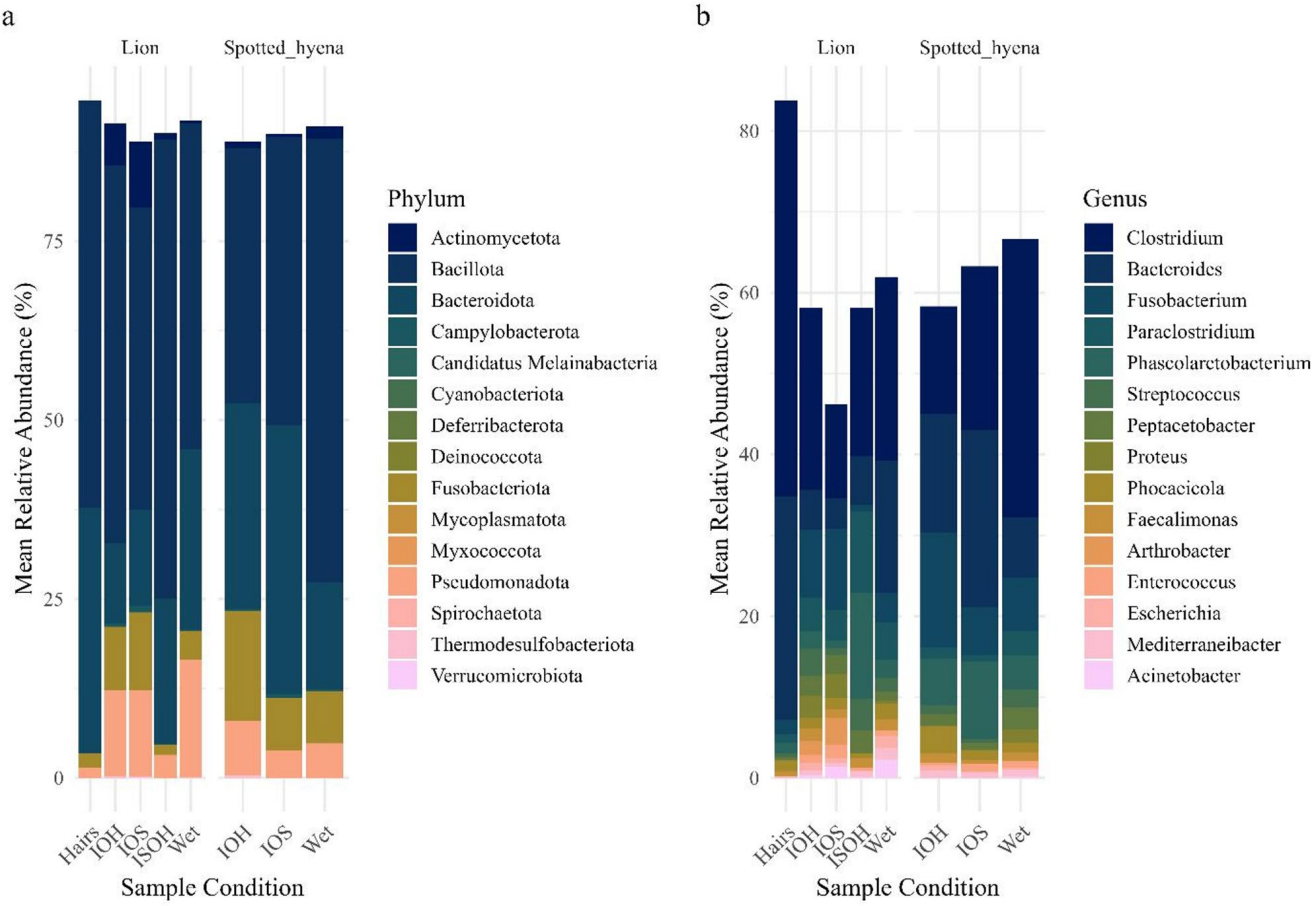
Comparison of (**a**) top bacterial phyla and (**b**) top genera by mean relative abundance, shown separately for African lion (*Panthera leo*) and spotted hyena (*Crocuta crocuta*) samples. Taxa shown represent the top 15 phyla and genera with > 1% mean relative abundance across all sample conditions. Sample conditions are denoted as “Wet” for fresh preserved samples, “IOH” for inside-outside hard, “IOS” for inside-outside soft, “ISOH” for inside soft/outside hard, and “Hairs” for samples with only hairs remaining. (**a**) The most abundant phyla included *Firmicutes* (*Bacillota*; 49% and 45%, respectively), *Bacteroidetes* (*Bacteroidota*; 15% and 25%), and *Fusobacteriota* (8% and 12%). (**b**) The most abundant genera were *Clostridium* (20% and 21%), *Fusobacterium* (8% and 11%, respectively), and *Bacteroides* (7% and 13%)

Across all sample conditions, the most abundant phyla, based on mean relative abundance, in lions and hyenas were *Firmicutes* (49% and 45%, respectively), *Bacteroidetes* (15% and 25%), *Fusobacteriota* (8% and 12%), *Proteobacteria* (13% and 6%), and *Actinomycetota* (5% and 1%) (Fig. 6a); the most abundant genera, based on mean relative abundance, were *Clostridium* (20% and 21%), *Fusobacterium* (8% and 11%, respectively), and *Bacteroides* (7% and 13%) (Fig. 6b). Additionally, *Paraclostridium* (4%) and *Streptococcus* (2%) were among the top 5 most abundant genera in lions, while *Phasolarctobacterium* (5%) and *Phocaeicola* (2%) were among the top 5 in hyenas. Variability in relative abundance among samples is illustrated in Fig. 6.

**Fig. 7:**
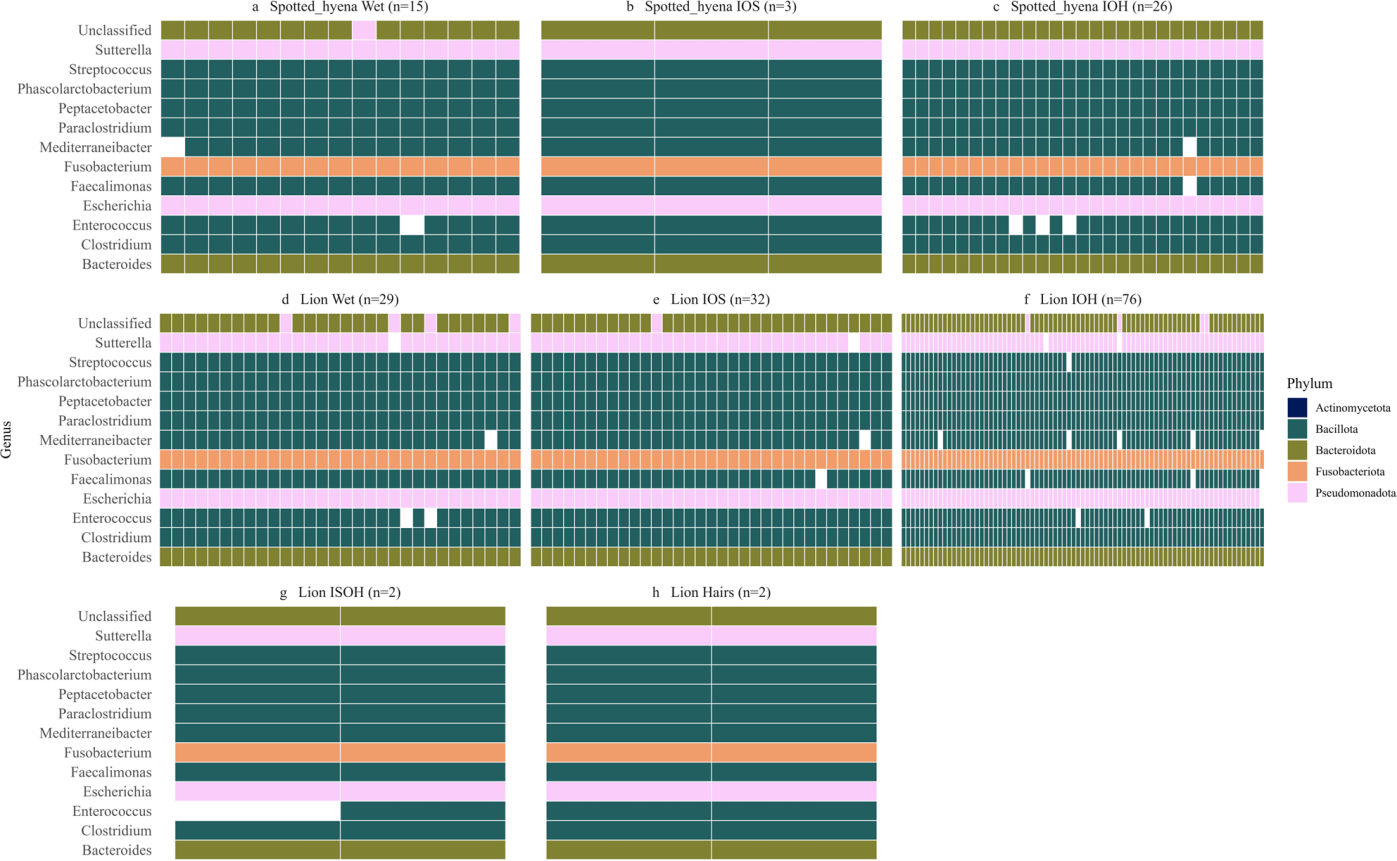
Binary heatmap of the top 1% most prevalent core (> 95%) bacterial genera across sample conditions for African lions (*Panthera leo*) and spotted hyenas (*Crocuta crocuta*). Genera were considered core if they appeared in ≥ 95% of samples within a group. Each tile represents the presence (colored) or absence (white) of a genus in an individual sample. Colors indicate bacterial phylum. Sample conditions are denoted as “Wet” for fresh preserved samples, “IOS” for inside/outside soft, “IOH” for inside/outside hard, “ISOH” for inside soft/outside hard, and “Hairs” for samples where only hair remained. Panels are ordered and labeled as follows: (**a**) Spotted hyena Wet (*n* = 15), (**b**) Spotted hyena IOS (*n* = 3), (**c**) Spotted hyena IOH (*n* = 38), (**d**) Lion Wet (*n* = 30), (**e**) Lion IOS (*n* = 33), (**f**) Lion IOH (*n* = 79), (**g**) Lion ISOH (*n* = 2), and (**h**) Lion Hairs (*n* = 2). Core host-associated genera such as *Clostridium*, *Fusobacterium*, and *Bacteroides* were consistently detected in all sample conditions

These genera also exhibited high prevalence across sample conditions, particularly in Wet, IOS, and IOH samples, indicating strong representation of core (≥ 95% prevalence), host-associated anaerobes. In Wet samples, *Clostridium* (27%), *Bacteroides* (13%), *Fusobacterium* (4%), *Paraclostridium* (4%), *and Phasolarctobacterium* (3%) dominated in mean relative abundance across lion and hyena samples and were present in 100% of samples along with *Escherichia*,* Faecalimonas*,* Peptacetobacter*,* and Streptococcus*. *Clostridium* (12%), *Fusobacterium* (10%), *Bacteroides* (5%), and *Paraclostridium* (4%) were among the most abundant taxa and exhibited 100% prevalence in IOS samples, suggesting these samples retained a strong host-microbiome signature. IOH samples exhibited similar profiles, with 100% prevalence of *Clostridium* (20%), *Fusobacterium* (10%), *Bacteroides* (7%), *Paraclostridium* (3%), *and Phasolarctobacterium* (3%). While Hairs (lion *n* = 2, hyena *n* = 0) and ISOH (lion *n* = 2, hyena *n* = 0) sample types were not included in statistical comparisons due to limited replication, their core microbiome profiles were explored to assess whether they retained key host-associated bacterial taxa. These samples consistently harbored core host-associated genera such as *Clostridium* and *Bacteroides*, but taxa like *Mediterraneibacter* and *Sutterella* also exhibited 100% prevalence, a pattern likely inflated by the stochastic effects of low sample size. Visualization with a binary heatmap (Fig. 7) revealed taxonomic overlap among Wet, IOS, IOH, ISOH, and Hairs samples, revealing that the majority of taxa were shared across all sample conditions, though each group also contained unique taxa.

**Fig. 8:**
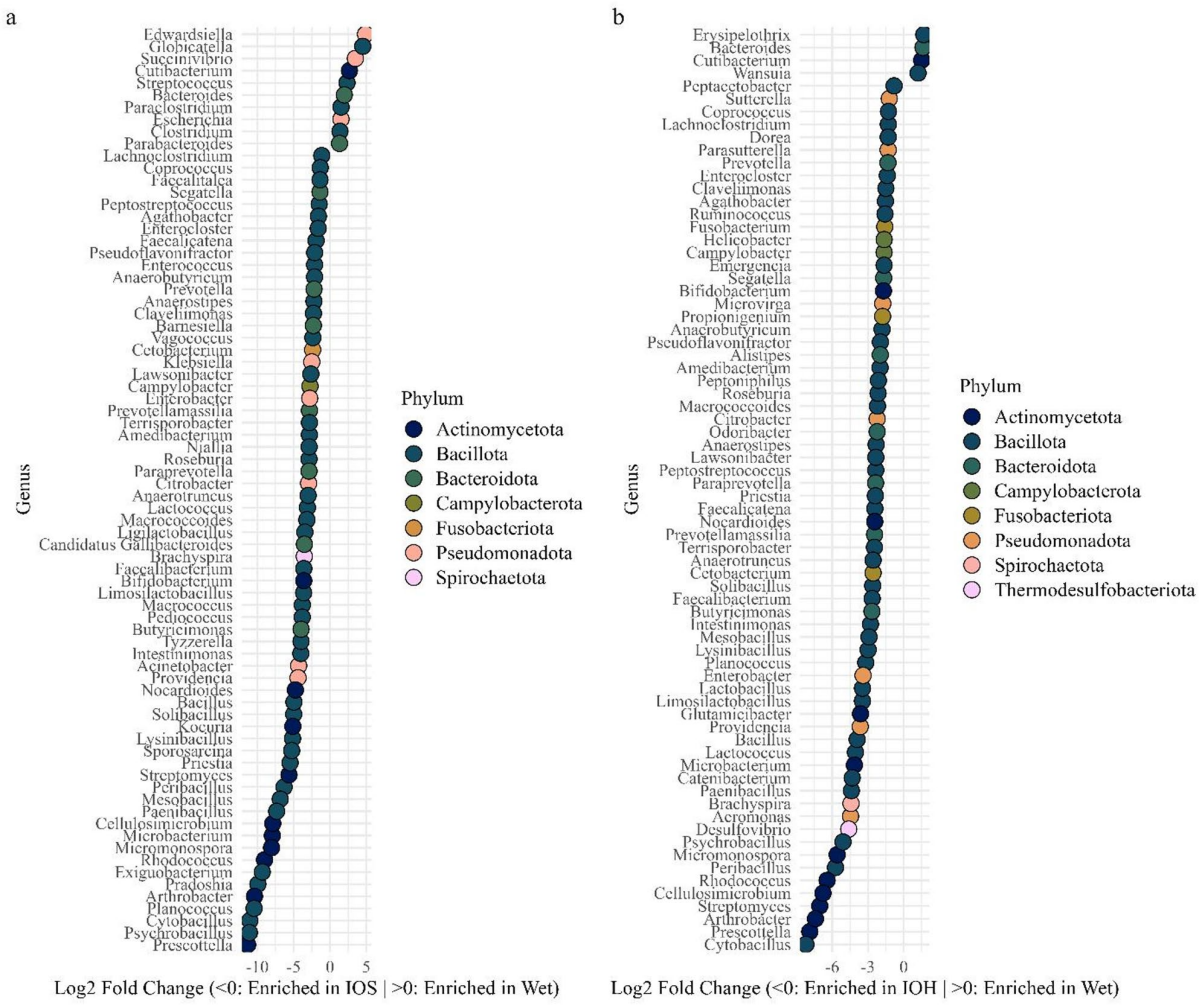
Differential abundance of significantly enriched bacterial genera identified by DESeq2 across African lions (*Panthera leo*) and spotted hyenas (*Crocuta crocuta*) in (**a**) Wet vs. IOS and (**b**) Wet vs. IOH comparisons. Sample condition identification is represented by “Wet” for fresh preserved samples, “IOS” for inside/outside soft samples, and “IOH” for inside/outside hard samples. Only genera with adjusted p-values < 0.050 (padj) are shown. Effect size is represented as log₂ fold change, where negative values indicate enrichment in IOS or IOH samples, and positive values indicate enrichment in Wet samples. (**a**) In the Wet vs. IOS comparison, 76 genera were significantly enriched, but none exceeded the 1% relative abundance threshold within their enriched group, suggesting statistical significance was primarily driven by low abundance or near absence in the comparison group. (**b**) In the Wet vs. IOH comparison, 72 genera were significantly enriched, but only *Prevotellamassilia*, *Solibacillus*, *Fusobacterium*, *Peptacetobacter*, and *Sutterella* exceeded 1% mean relative abundance within the group in which it was enriched (IOH), indicating biologically meaningful enrichment

The Wet vs. IOS (Fig. 8a) comparison identified 10 enriched genera (padj < 0.05) within Wet samples, including *Edwardsiella*, *Globicatella*, *Succinivibrio*, and *Cutibacterium*, while 66 enriched genera (padj < 0.05) were identified within IOS samples, including *Prescottella*, *Psychrobacillus*, *Cytobacillus*, and *Planococcus*. Genera are listed in order of effect size, from greatest to smallest log₂ fold change within each group (i.e., most positive in Wet and most negative in IOS). However, none of these exceeded 1% mean relative abundance within their enriched group, suggesting that statistical differences were driven by low or near-zero presence in one group rather than dominance in the other. In contrast, in the Wet vs. IOH comparison (Fig. 8b), 4 enriched genera (padj < 0.05) were identified within Wet samples, including *Erysipelothrix*, *Cutibacterium*, *Bacteroides*, and *Wansuia*, while 68 enriched genera (padj < 0.05) were identified within IOH samples, including *Cytobacillus*, *Prescottella*, *Arthrobacter*, and *Streptomyces*. Of the genera enriched in IOH samples relative to Wet samples, *Solibacillus* (2%), *Prevotellamassilia* (3%), *Fusobacterium* (17%), *Sutterella* (10%), *and Peptacetobacter* (13%) were the only taxa to exceed 1% mean relative abundance in IOH samples. Comparison of DESeq2-identified enriched genera revealed substantial overlap between IOS- and IOH-enriched taxa, with 50 of 66 IOS-enriched genera also enriched in IOH samples, while 16 genera were unique to IOS and 18 were unique to IOH. Host-stratified DESeq2 analyses identified multiple differentially abundant genera in lions for both Wet vs. IOS and Wet vs. IOH comparisons (Additional file 2). In hyenas, only a small number of genera were identified as differentially abundant in either comparison (Additional file 3).Complementary differential abundance testing using ANCOM-BC2 identified several genera (*Coprococcus*, *Dorea*, *Faecalicatena*, *Lachnoclostridium*, *Peptostreptococcus*, *Prevotellamassilia*, and *Cetobacterium*) as differentially abundant between Wet and IOH samples under the primary model; however, none of these taxa passed the sensitivity analysis for pseudo-count addition, indicating that these differences were not robust to alternative zero-handling assumptions. No genera were identified as differentially abundant between Wet and IOS samples using ANCOM-BC2.

## Discussion

This study aimed to evaluate how fecal storage techniques and sample conditions correlate with variation in GMB diversity and composition in two large African carnivores under field conditions. Although immediate preservation of fecal samples at the time of defecation remains the gold standard for microbiome studies [7], ambient drying of fecal material has been used in wildlife molecular ecology as a practical field method for preserving DNA when freezing or preservation reagents are not feasible [15]. Additionally, studies have demonstrated that fecal microbiome composition and diversity metrics remain reproducible during short-term storage at ambient temperature, supporting the feasibility of field-based sampling when immediate preservation is not possible [16]. By comparing fresh fecal samples preserved in validated PERFORMAbiome animal GMB DNA collection tubes, which contain a proprietary stabilization solution with < 24% ethanol and have been shown to preserve wildlife fecal microbiome composition comparably to immediate freezing during ambient storage [17], to those dried without preservatives, we found that both preservation methods maintained gut microbiome diversity and composition under short-term, semi-arid field conditions, whereas Opportunistic samples, though retaining core taxa, showed environmentally driven compositional shifts, underscoring the need for consistent sampling protocols. The results provide important context for interpreting GMB data from conservation field studies, especially when logistical constraints necessitate varied collection or storage methods.

### Storage technique

Our comparison of GMBs in Wet versus Dry fecal samples collected during animal captures revealed no differences in alpha or beta diversity, suggesting overall stability in microbiome community structure across these storage techniques. While taxon-specific differences were detected using count-based differential abundance analysis, such as the enrichment of *Succinivibrio* in Dry samples and *Rummeliibacillus* in Wet samples, none of the taxa enriched in Wet samples exceeded 1% mean relative abundance. In several cases, the mean abundance of enriched taxa was near or below detection thresholds. These findings suggest that differential abundance results were influenced primarily by low-abundance or inconsistently detected taxa rather than consistent shifts in dominant community members, underscoring the importance of considering both statistical significance and ecological relevance in microbiome comparisons. To evaluate the robustness of these findings under a compositional framework, we additionally applied ANCOM-BC2, which corrects for bias due to unequal sampling fractions and is generally more conservative than count-based approaches. ANCOM-BC2 identified only two genera as differentially abundant: *Succinivibrio*, enriched in Dry samples, and *Campylobacter*, enriched in Wet-preserved samples. The reduced number of significant taxa detected by ANCOM-BC2 supports the interpretation that most storage-associated differences identified by count-based methods reflect low-abundance or sporadically detected taxa rather than consistent shifts in dominant community composition.

Although differences were detected in specific taxa, particularly among those with low relative abundance, overall microbiome richness and the composition of dominant taxa did not differ between storage techniques. These findings suggest that both storage techniques, immediate preservation in stabilization media and air-drying overnight in paper bags, are suitable for maintaining GMB community diversity and composition in short-term field conditions in semi-arid environments with high evaporation, such as Etosha. Because samples were collected fresh and a subset of the initial, whole fecal mass was dried in brown paper bags immediately, the consistency in diversity metrics reflects the minimal impact of fast, short-term drying when desiccation is rapid and contamination risk is low. This has important implications for fieldwork in remote or resource-limited settings, where access to preservatives or refrigeration may be constrained. Drying fecal samples in clean paper bags represents a practical, low-cost alternative that preserves microbiome community diversity and composition sufficiently for broad ecological assessments, such as comparisons of alpha and beta diversity or relative abundance of dominant taxa. However, this method may be less suitable for assessments of functional potential via metagenomics or transcriptomics, which may be more sensitive to degradation or compositional shifts introduced by desiccation. Future studies may still assess functional potential and strain-level or subspecies variation that reflect different genotypic or phenotypic traits. Such analyses may be more sensitive to storage differences, but our results support the interchangeability of these storage methods for broad ecological assessments of carnivore GMB communities.

### Sample condition

Comparisons involving Wet samples and opportunistically collected IOH and IOS samples revealed more pronounced differences in diversity metrics. Alpha diversity was significantly reduced in IOH and IOS samples compared to Wet samples, and beta diversity analyses detected differences in community composition, particularly between Wet and IOS samples, characterized by increased dispersion and variability in IOS samples rather than distinct clustering, as supported by PERMANOVA results and NMDS ordinations. The substantial overlap observed between some IOS and Wet samples may reflect opportunistic samples collected shortly after defecation, prior to extensive environmental exposure. In contrast, the broader dispersion of IOS samples likely reflects variable exposure duration and microenvironmental conditions, contributing to increased heterogeneity in community membership. Because opportunistic samples varied in both estimated time since defecation and environmental exposure prior to collection, these factors cannot be fully disentangled in the present study. DESeq2 analysis identified 68 and 66 genera significantly enriched in IOH and IOS samples, respectively. Notably, the majority of these taxa overlapped between sample conditions, with 50 genera enriched in both IOS and IOH samples. This substantial overlap indicates that taxa enriched in IOS samples generally remain enriched at the IOH stage, consistent with progressive amplification of environmentally associated taxa with increasing exposure duration rather than complete taxonomic turnover. However, a smaller subset of genera was unique to each condition, suggesting that some taxa may preferentially proliferate or decline at later stages of environmental exposure. When analyses were stratified by host species, differential abundance patterns were more pronounced in lions than in spotted hyenas. Numerous genera were detected in lions across both Wet vs. IOS and Wet vs. IOH contrasts, whereas few genera were identified in hyenas, suggesting that the hyena gut microbiome was less sensitive to variation in sample condition. While differences in presence or absence can be ecologically meaningful, particularly for functionally important taxa, the majority of differentially abundant genera identified in this study were low in both relative abundance and prevalence. In contrast, core taxa (defined as those detected in ≥ 95% of samples per group) remained consistently represented across sample types, suggesting that community shifts were primarily driven by less dominant taxa. Although abundance does not necessarily equate to functional importance, and rare taxa can exert critical ecological roles, our findings suggest that differences in sample condition are associated with variation in the detection of low-abundance or environmentally sensitive organisms. Reductions in obligate anaerobes in opportunistically collected feces have been reported with increasing time since deposition under field conditions, consistent with post-defecation exposure driving compositional shifts rather than reflecting true absence from the gut community [6]. For instance, the reduced prevalence of obligate anaerobes such as *Paraclostridium* and *Fusobacterium* in IOH samples likely reflects oxygen exposure post-defecation rather than absence from the gut microbiome. Thus, while Opportunistic samples retain core community members, variation introduced by environmental degradation must be considered when interpreting compositional differences. This highlights the importance of consistent sampling protocols when comparing gut microbiomes.

Our findings align with previous research demonstrating the resilience of the GMB community structure under certain short-term environmental exposures. Minimal shifts in dominant phyla were observed during early post-defecation intervals in giraffes and springbok [6], and cheetah microbiome composition remained stable over several days under dry conditions [12]. Likewise, studies in elk and other large mammals suggest that the microbiome may remain relatively stable up to a week in temperate climates during the winter [10]. Our results reinforce these findings and further extend them to African carnivores, demonstrating that rapid drying in a semi-arid environment can maintain core gut bacterial signatures. However, our findings contrast with studies from more humid environments, such as tropical regions of China where significant shifts in GMB diversity and composition were observed in spider monkey fecal samples after 24 h [11]. This underscores that environmental context, particularly humidity, is a critical determinant of sample stability and preservation success.

While this study demonstrates that desiccated field samples can yield microbiome profiles similar to preserved samples in semi-arid environments, methodological consistency remains critical. Including both fresh samples collected directly from the host (whether chemically preserved or dried immediately) and opportunistically collected ground samples in the same analysis may introduce artificial variation. Additionally, differences in DNA extraction protocols, such as the use of a brief heat step to improve DNA yield from Dry and Opportunistic samples, represent a further source of potential methodological variability. This heat step was applied consistently across all non-Wet samples to maximize DNA recovery from potentially degraded material; however, its effects cannot be fully disentangled from sample condition and are therefore acknowledged as a limitation of this study. GMB profiles differed significantly in common cranes depending on whether samples were collected noninvasively or from trapped and sedated individuals [9]. The differences observed in common cranes were not fully explained by environmental contamination, suggesting that sample collection context, including stress, sedation, or time to defecation, can influence GMB composition. Therefore, for comparative GMB analyses, researchers should ideally employ a single sampling strategy across all individuals or separate analyses by sampling method. Future research should prioritize time series and field-based desiccation studies across diverse host species and environments to evaluate how sample collection methods may affect GMB diversity and composition. These studies would help determine whether differences in sampling strategy introduce systematic bias into comparative GMB analyses. Broader adoption of desiccation-based methods could improve accessibility and standardization of GMB research across conservation projects, especially where traditional preservation methods are logistically impractical.

## Conclusions

Our findings demonstrate that simple, low-cost desiccation methods can preserve gut microbiome diversity in African carnivores’ fecal samples under semi-arid field conditions, supporting the broader use of such approaches in wildlife research where traditional preservation methods are impractical. Core bacterial communities remained stable despite opportunistic sampling and environmental exposure, underscoring the resilience of dominant taxa. Adoption of standardized, accessible sampling protocols will expand opportunities for comparative microbiome studies across diverse habitats and species, advancing both ecological and conservation research.

## Methods

### Study area

This study was conducted in Etosha National Park (ENP) in northern Namibia, a semi-arid savanna ecosystem characterized by frequent droughts, high evaporation, and strong spatial variation in rainfall from west to east, resulting in a heterogeneous pattern of consumer resources [13, 18]. Mean annual rainfall ranges from approximately 250–600 mm, falling predominantly during the November–April wet season, while the May–October dry season is marked by low humidity, minimal precipitation, and high solar radiation [13, 18]. Mean annual temperatures exceed 22 °C across most of the basin, with some of the highest temperatures in the country occurring during the early summer months [18]. Relative humidity varies seasonally, averaging over 80% in March (late wet season) but frequently dropping below 20% in September during the dry season [18].

### Sample collection

#### Storage technique

To assess the validity of storage techniques, fresh fecal samples were collected from individual African lions (P*anthera leo*, *n* = 30) and spotted hyenas (*Crocuta crocuta*, *n* = 15) while anesthetized as part of ongoing studies on the spatial ecology of these species in Etosha National Park [19]. Briefly, animals were chemically immobilized via vehicle-based darting using bait and call-ins under standard protocols; when available, fecal material was collected during handling. All immobilizations were performed by veterinarians registered with the Namibian Veterinary Council and the Ministry of Environment, Forestry, and Tourism in accordance with University of Georgia and Northern Michigan University approved IACUC proposals (A2021 04-013-42-A7 and RCIV00072018, respectively). A subsample (approximately 630 mg, per manufacturer’s protocol) of each whole fecal mass was preserved in PERFORMAbiome animal GMB DNA collection tubes (DNA Genotek Inc.) which contain a proprietary stabilization solution with < 24% ethanol and stored in a refrigerator (hereafter referred to as Wet). These tubes have been previously validated for wildlife microbiome research, demonstrating preservation of microbial community composition comparable to immediate freezing and stability during extended ambient storage [17]. When sufficient material remained, an additional subsample (5–10 g) from a subset of lion (*n* = 16) and hyena (*n* = 4) fecal samples was placed in brown paper bags and suspended outdoors under ambient temperature and humidity for at least 24 h to dry before being transferred to screw-cap containers for storage in ambient temperature (hereafter referred to as Dry). Samples were collected between May 2022 and June 2024 and shipped at ambient temperature from Namibia to the United States. Upon arrival, Dry samples were stored at room temperature, and Wet samples were stored in a -80° freezer before DNA extraction.

### Sample condition

**Fig. 9:**
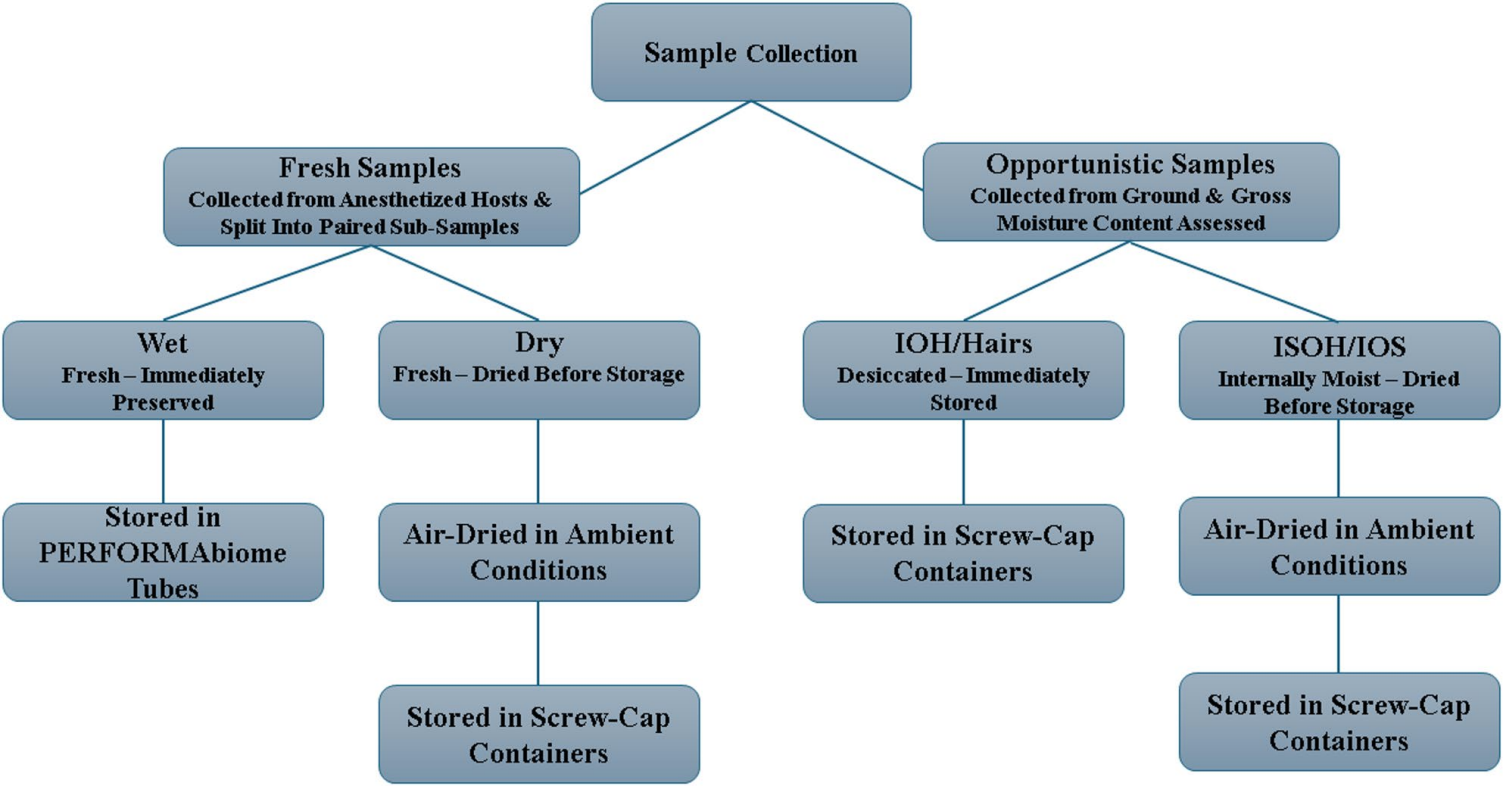
Schematic illustrating fecal sample collection pathways, moisture assessment, and storage treatments. Fecal samples were obtained from free-ranging lions (*Panthera leo*) and spotted hyenas (*Crocuta crocuta*) in Etosha National Park, Namibia. Fresh samples were collected directly from anesthetized hosts and divided into paired sub-samples. One sub-sample was immediately preserved in PERFORMAbiome tubes (“Wet”), while the paired sub-sample was air-dried under ambient field conditions prior to storage in screw-cap containers (“Dry”). Opportunistic samples were collected from the ground along roads, around waterholes, or near carnivore kill sites and gross moisture content was assessed at the time of collection. Samples classified as internally moist [inside/outside soft (“IOS”) and inside soft/outside hard (“ISOH”)] were air-dried under ambient conditions before storage in screw-cap containers. Samples classified as desiccated [inside/outside hard (“IOH”) and only hairs remaining (“Hairs”)] were stored directly in screw-cap containers without additional drying

To assess the impact of sample condition on GMB diversity and composition, Wet samples were compared to a set of lion (*n* = 116) and hyena (*n* = 41) fecal samples collected opportunistically off the ground (hereafter, Opportunistic) along roads, around waterholes, or near carnivore kill sites within our study area. Carnivore host species were initially assigned from fecal morphology (e.g. size, shape, texture), and the gross moisture content was recorded. Gross moisture content was recorded only for opportunistically collected samples, as fresh samples collected directly from anesthetized animals were immediately subsampled and preserved. Host identity was subsequently verified via DNA metabarcoding of the same samples conducted by Jonah Ventures (Patterson, 2025); these data were used solely to confirm field assignments and were not included in the analyses reported here. Samples not identified to be African lion or spotted hyena were excluded from this study, and all reported sample counts reflect only confirmed host identifications. Gross moisture content was assessed by breaking open the fecal mass with gloved hands to inspect internal and external consistency before subsampling (5–10 g) into screw-cap containers for storage. Moisture content categories for Opportunistic samples included inside and outside soft (IOS), inside soft and outside hard (ISOH), and inside and outside hard (IOH). Some samples were degraded to the point that only hairs remained (Hairs). Opportunistic samples that maintained moisture (i.e., IOS or ISOH) were placed in brown paper bags and hung in trees to dry overnight, using the same ambient field-drying approach applied to Dry samples, before placement into screw cap containers for storage. A schematic of the sampling workflow is illustrated in Fig. 9. Time since fecal deposition was unknown for all samples, and no controlled field aging experiments were conducted. However, based on field observations and condition classifications, IOS samples were estimated to be less than 1 day old, ISOH less than 3 days old, IOH approximately 1 week or older, and Hairs likely over 1 month old. Opportunistic samples were collected between October 2021 and March 2024, stored at room temperature from collection until arrival to the United States in August 2025 for DNA extraction.

### DNA extraction and sequence processing

DNA was extracted from Wet feces using DNeasy PowerSoil Pro DNA Kits [20]. An additional heat step was added to the QIAGEN protocol for all Dry and Opportunistic samples to obtain a sufficient quantity of DNA from potentially degraded samples [21]. Specifically, samples were heated for 10 min at 65 degrees Celsius before the first vortex step to improve DNA yield [22]. The decision to add a heat step was supported by previous research indicating that the extraction technique influences DNA yield and that storage media, but not the extraction method, influence the diversity and abundance of microbiome communities recovered [21, 23]. The target amount of homogenized fecal material used for DNA extraction was 200 µl for Wet samples and 250 milligrams for Dry and Opportunistic samples. Aliquots in equimolar ratios were sent to Argonne National Laboratory (ANL) for paired-end shallow shotgun metagenomic sequencing on the Illumina NextSeq 2000 platform to read genomic DNA to identify gut bacteria. ANL includes positive and negative controls in each sequencing run and proceeds only if negative controls are free of contamination and positive controls yield expected results, ensuring sequencing integrity. FASTQ files received from the ANL were uploaded to the Galaxy [24] Europe server to join sequences by creating a paired-end library and quality filter using FASTQC [25] and TRIMMOMATIC [26]. The Galaxy Europe instance was selected for taxonomic classification in this study due to its increased memory capacity and early integration of the Kraken2 nt_core reference database [27]. While Kraken2 is available through other pipelines such as QIIME2, the nt_core database is substantially larger than the standard Kraken2 database and can be difficult to install and run locally. FASTQC was used to assess the quality of sequences before and after trimming and filtering reads. Paired-end sequences were trimmed with the sliding window set to 4, minimum length set to 50 base pairs, and head crop set to 13 base pairs to reduce noise. Nextera standard adapter sequences were also removed. Kraken2 [27] was used to classify bacterial reads. The confidence score (CS) of 0.4 and minimum hit group of 3 were used to reduce false positives identified when using the default minimum hit group setting (2) and CS (0.2) and to improve sensitivity resulting from an increased CS of 0.6 [28]. Although species-level assignments were explored by adjusting confidence thresholds, classification confidence at the species level was inconsistent across taxa; therefore, downstream analyses were conservatively conducted at the phylum and genus levels. After quality filtering and taxonomic classification, sequencing depth per sample ranged from 1,755 to 222,041 reads (median = 52,444; mean = 75,422). A read-based taxonomic classification approach was used rather than assembly-based methods. A read-based taxonomic classification approach was used rather than assembly-based methods. Shallow shotgun metagenomic sequencing is effective for profiling microbial community composition, but sequencing depth strongly influences the recovery of genomic content and the reconstruction of metagenome-assembled genomes (MAGs) [3]. While relatively low sequencing depth can capture broad taxonomic patterns, de novo assembly from complex gut microbiomes requires substantially greater coverage, and MAG recovery is strongly dependent on sequencing depth and community complexity [3, 29]. In this study, sequencing depth per sample (median ≈ 52,000 reads) was well below thresholds typically required for reliable assembly [29], and assembly-based approaches at low sequencing depths can bias results toward highly abundant taxa and reduce comparability across samples [30]. Because the primary objectives of this study were to assess gut microbiome diversity and taxonomic composition across sample storage and condition, a read-based approach was selected as the most appropriate and conservative analytical strategy. Community composition across all samples was visualized using Pavian [31]. The Kraken software suite [32] was used to create a Biological Information Matrix (BIOM) [33] file and an OTU ID file from the filtered tabular Kraken output reports in Galaxy. The “phyloseq” [34] package was used to convert the BIOM file into a phyloseq object within Galaxy. To construct a phylogenetic tree for diversity analyses, we extracted taxonomic lineage information from the OTU ID file generated by the Kraken to BIOM tool using TaxonKit (v0.12.0), mapping each taxonomic identifier to its complete taxonomic lineage based on the NCBI taxonomy reference database. Taxa were filtered to retain only bacterial lineages for tree construction and statistical analysis. The resulting taxonomy table was used to construct a taxonomic tree in Newick format using the Python ETE Toolkit (v3.1.2) with the “anytree” package. This tree represents hierarchical taxonomic relationships rather than inferred phylogeny. Sample observation metadata and the Newick tree were then added to the phyloseq object for diversity analyses in RStudio.

### Statistical analysis

#### Storage technique

Paired samples (e.g. Wet and Dry) from lions (*n* = 16) and hyenas (*n* = 4) were compared to evaluate the effects of storage technique on GMB diversity and composition. For scaling with rank subsampling [35], samples were normalized to 5,000 reads based on rarefaction curves (see Additional file 4), establishing this value as the minimum sequencing depth (Cmin) to ensure sufficient coverage for community-level analysis while maximizing sample retention. One pair of lion samples was excluded from the analyses due to insufficient sequencing depth (< 5000 reads). Alpha diversity metrics (Shannon, Observed, Simpson’s, Chao1, and Faith’s PD) were calculated. Shapiro–Wilk tests of the differences between paired Wet and Dry samples (*p* > 0.050 for all metrics) supported the use of parametric paired t-tests for alpha diversity comparisons. Relative abundance was visualized at the phylum and genus levels. Beta diversity was assessed using Bray–Curtis dissimilarities (based on species abundance) and UniFrac distances (weighted and unweighted) calculated from a taxonomy-derived tree constructed using the NCBI taxonomic hierarchy. Because this tree lacks branch lengths based on sequence divergence, the UniFrac results reflect taxonomic relatedness rather than true phylogenetic distance and should be interpreted with caution. A PERMDISP test (F = 0.249, *p* = 0.062) supported the use of a non-parametric PERMANOVA test for differences between Wet-Dry pairs. To complement these approaches and explicitly account for compositionality, beta diversity was additionally evaluated using Aitchison distance (Euclidean distance on centered log-ratio–transformed counts), which does not require rarefaction [36]. Differential abundance analyses were conducted using both DESeq2 [37] and Analysis of Compositions of Microbiomes [38] with Bias Correction (ANCOM-BC2) to assess robustness to analytical approach. DESeq2 was applied to paired samples to identify taxa enriched by storage condition (adjusted *p* < 0.05), and mean relative abundance (RAB) was calculated across and within storage techniques. DESeq2 results were further filtered to highlight major taxa with a mean RAB greater than 1% overall and within respective groups. Additional differential abundance analyses were performed with DESeq2 separately for lions and spotted hyenas to control for host effects.

### Sample condition

To investigate the influence of sample conditions on GMB composition (Table 1), alpha and beta diversity were assessed across Wet, IOS, ISOH, IOH, and Hairs samples within lions (*n* = 146) and hyenas (*n* = 45), as detailed below. Dry pairs were excluded from this analysis.

**Table 1:** Sample counts for each condition within lions (*Panthera leo*) and hyenas (*Crocuta Crocuta*)

Sample condition	African lion (n)Before/after Scaling (Ranked Subsampling)	Spotted hyena (n)Before/after Scaling (Ranked Subsampling)
Fresh Preserved (Wet)	30/29	15/15
Inside/Outside Soft (IOS)	33/32	3/3
Inside Soft/Outside Hard (ISOH)	2/2	0/0
Inside/Outside Hard (IOH)	79/76	27/26
Only Hairs Remaining (Hairs)	2/2	0/0

Values represent sample counts before and after scaling with ranked subsampling (SRS). IOS = inside–outside soft; ISOH = inside soft–outside hard; IOH = inside–outside hard; Hairs = only hairs remaining

To compare broader sample conditions (including Wet, IOS, IOH, ISOH, and Hairs), samples were normalized to 5,000 reads using scaling with ranked subsampling to ensure consistency with the Wet vs. Dry analysis. Rarefaction curves confirmed this threshold provided sufficient depth for reliable community-level comparisons while retaining all available samples (see Additional file 5). Three IOH, one IOS, and one Wet lion samples were removed due to insufficient sequencing depth (< 5000 reads), while one IOH hyena sample was removed due to insufficient sequencing depth (< 5000 reads) (Table 1). Bonferroni-corrected pairwise comparisons were used per diversity metric. Homogeneity of multivariate dispersions was assessed using PERMDISP, and no significant differences (F = 0.824, *p* = 0.532) in group dispersions were detected across sample conditions. ISOH (lion *n* = 2, hyena *n* = 0) and Hairs (lion *n* = 2, hyena *n* = 0) were excluded from PERMANOVA due to insufficient sample size. Differential abundance analyses across sample conditions were conducted using both DESeq2 and ANCOM-BC2. To control for host-specific microbiome differences, additional models were performed separately for lions and spotted hyenas. Mean RAB was calculated within each sample condition. A binary heatmap was used to visualize shared and condition-specific taxa presence across sample conditions. Core taxa were defined as those present in more than 95% of samples within each sample condition group. To highlight the most dominant members of the core microbiome, the top 1% most prevalent taxa were identified for each sample condition based on their frequency of occurrence across samples. Visualizations (e.g. binary heatmap) were grouped by sample condition, and taxonomic identities were determined via Genus-level annotations.

## Supplementary Information

Below is the link to the electronic supplementary material.

Supplementary Material 1: Additional file 1. DESeq2 differential abundance results for storage technique in lions (PDF). Log2 fold-change plot showing genera identified as significantly differentially abundant between Wet and Dry lion fecal samples using DESeq2. Negative values indicate genera enriched in Wet samples, and positive values indicate genera enriched in Dry samples. Only taxa remaining significant after multiple-testing correction are shown.

Supplementary Material 2: Additional file 2. Host-stratified DESeq2 differential abundance results for lions (PDF). Genus-level differential abundance analysis comparing Wet vs. IOS (panel a) and Wet vs. IOH (panel b) samples in lions. Points represent significantly differentially abundant genera (adjusted *p* < 0.05), colored by phylum. Log2 fold change values indicate enrichment relative to Wet samples (negative values indicate enrichment in IOS or IOH; positive values indicate enrichment in Wet).

Supplementary Material 3: Additional file 3. Host-stratified DESeq2 differential abundance results for spotted hyenas (PDF). Genus-level differential abundance analysis comparing Wet vs. IOS (panel a) and Wet vs. IOH (panel b) samples in spotted hyenas. Points represent significantly differentially abundant genera (adjusted *p* < 0.05), colored by phylum. Log2 fold change values indicate enrichment relative to Wet samples (negative values indicate enrichment in IOS or IOH; positive values indicate enrichment in Wet).

Supplementary Material 4: Additional file 4. Rarefaction curves for storage technique comparison (PDF). Rarefaction curves showing sequencing depth for all samples included in the storage technique analysis. Vertical dashed lines indicate the selected rarefaction threshold (5,000 reads) and the 10th percentile sequencing depth (7,885 reads).

Supplementary Material 5: Additional file 5. Rarefaction curves for sample condition comparison (PDF). Rarefaction curves showing sequencing depth for samples included in the sample condition analysis. Vertical dashed lines indicate the selected rarefaction threshold (5,000 reads) and the 5th percentile sequencing depth (9,022 reads).

## Data Availability

Raw sequencing data have been deposited in the NCBI Sequence Read Archive under [BioProject accession PRJNA1289876] (https://zenodo.org/records/15746479?preview=1%26token=eyJhbGciOiJIUzUxMiJ9.eyJpZCI6ImY3YmJkMWYyLTEzN2YtNGJlZi1hOGE5LWU2ZTc2ZTRjNmY5YSIsImRhdGEiOnt9LCJyYW5kb20iOiI1MjQ4MGZhNTM0NDUyYjY2MWE4NjQ0MjhkMjZjNWI4OSJ9.oFnTVhU5wMLnJkeQq5HrNgbF8V_lh_pCOhEgxqTyn-pmW7VX6_PVCVatkgSzyzOBj5JHFF3UZWNNoC2H4D2T_g) and are accessible to reviewers only.All processed data, analysis code, and workflow documentation are available to reviewers in a [Zenodo repository] (https://zenodo.org/records/15746479?preview=1%26token=eyJhbGciOiJIUzUxMiJ9.eyJpZCI6ImY3YmJkMWYyLTEzN2YtNGJlZi1hOGE5LWU2ZTc2ZTRjNmY5YSIsImRhdGEiOnt9LCJyYW5kb20iOiI1MjQ4MGZhNTM0NDUyYjY2MWE4NjQ0MjhkMjZjNWI4OSJ9.oFnTVhU5wMLnJkeQq5HrNgbF8V_lh_pCOhEgxqTyn-pmW7VX6_PVCVatkgSzyzOBj5JHFF3UZWNNoC2H4D2T_g).

## Declarations

### Disclaimer

This manuscript was prepared as an account of work sponsored by an agency of the United States Government. Neither the United States Government nor any agency thereof, nor any of their employees, makes any warranty, express or implied, or assumes any legal liability or responsibility for the accuracy, completeness, or usefulness of any information disclosed, or represents that’s its use would not infringe privately owned rights. Reference herein to any specific commercial product, process, or service by trade name, trademark, manufacturer, or otherwise does not constitute or imply its endorsement, recommendation, or favoring by the United States Government or any agency thereof. The views and opinions of the authors expressed herein do not necessarily state or reflect those of the United States Government or any agency thereof.

## Abbreviations

GMB: Gut microbiome
PCR: Polymerase chain reaction
Wet: Freshly preserved samples
Dry: Samples dried for 24 h under ambient conditions
Opportunistic: Samples collected from the ground at roads, waterholes, or carnivore kill sites
IOS: Inside–outside soft
ISOH: Inside soft–outside hard
IOH: Inside–outside hard
Hairs: Degraded samples containing only hairs
NMDS: Non-metric multidimensional scaling
CS: Confidence score
BIOM: Biological observation matrix
Cmin: Minimum sequencing depth
RAB: Relative abundance
