# Supplementary figures and images for: African carnivore gut bacterial diversity and composition are associated with sample condition but not storage technique
Source: Anim Microbiome. 2026 Mar 24;8:59. doi: 10.1186/s42523-026-00553-w (PMC13134297; doi:10.1186/s42523-026-00553-w)

## DESeq2 (Lion only): Wet vs Dry

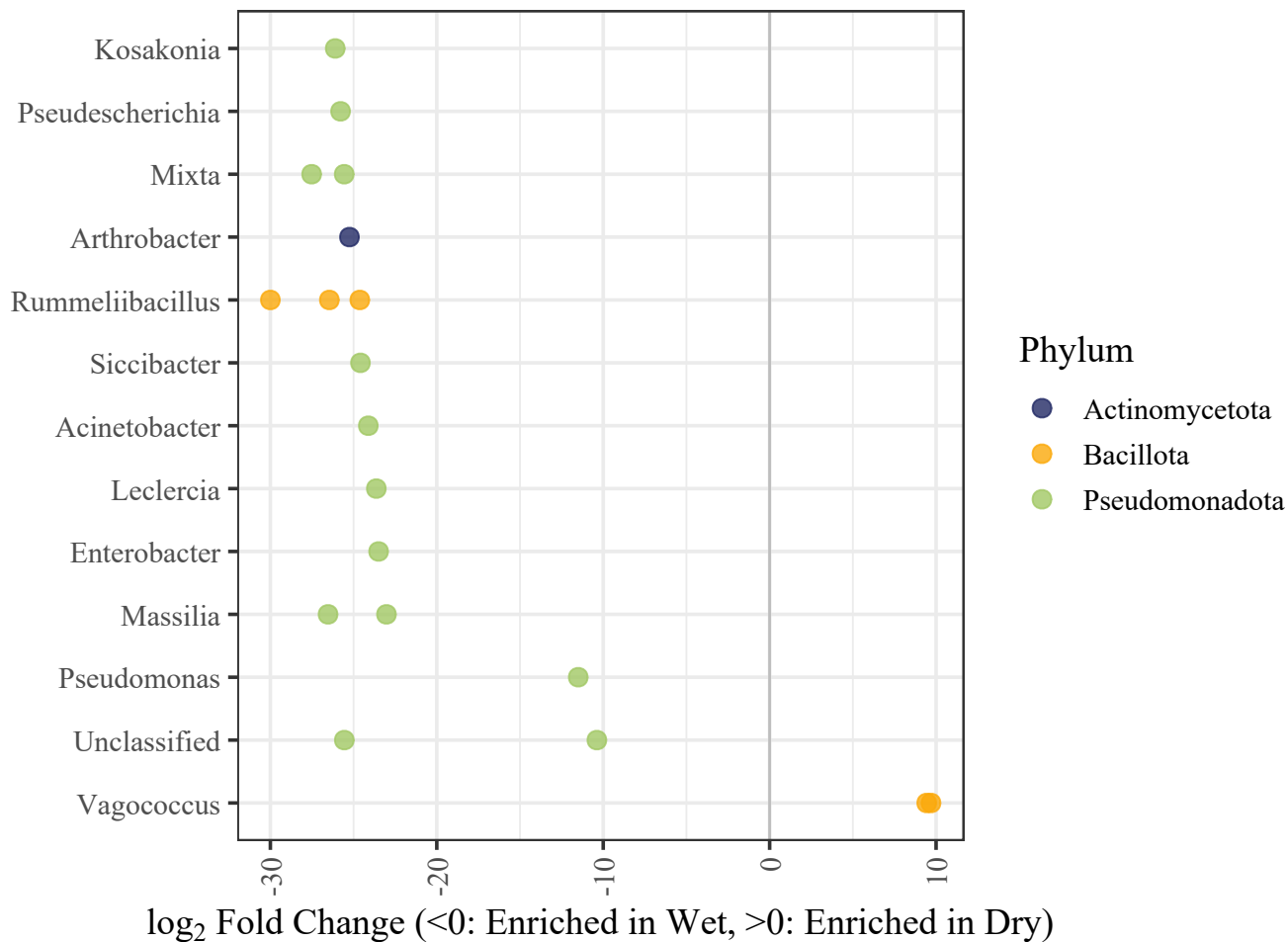

Supplement: Supplementary file 1 — Supplementary Material 1: Additional file 1. DESeq2 differential abundance results for storage technique in lions (PDF). Log2 fold-change plot showing genera identified as significantly differentially abundant between Wet and Dry lion fecal samples using DESeq2. Negative values indicate genera enriched in Wet samples, and positive values indicate genera enriched in Dry samples. Only taxa remaining significant after multiple-testing correction are shown. [file 42523_2026_553_MOESM1_ESM.pdf]

DESeq2 differential abundance by sample condition (Lion)

a

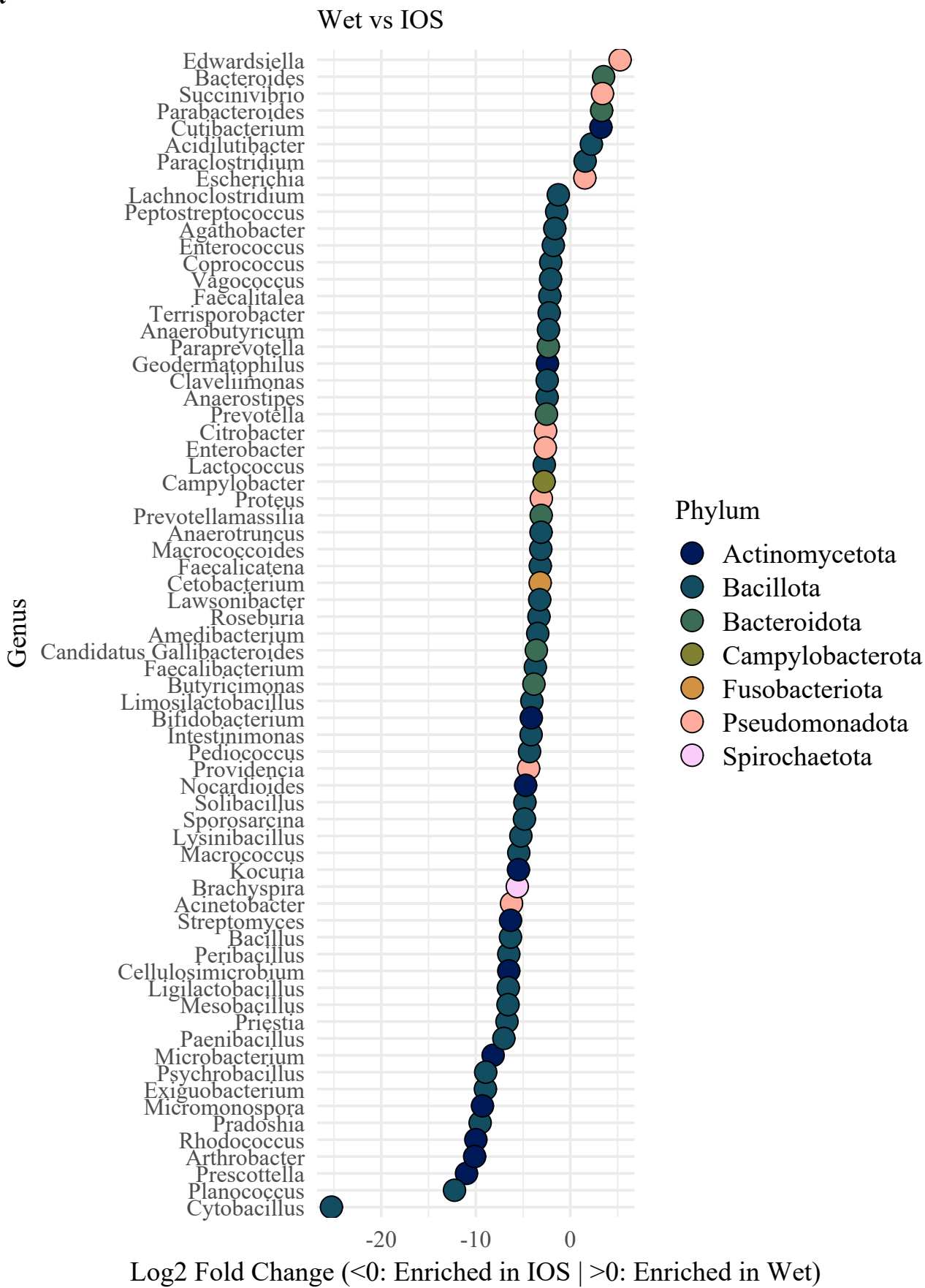

b

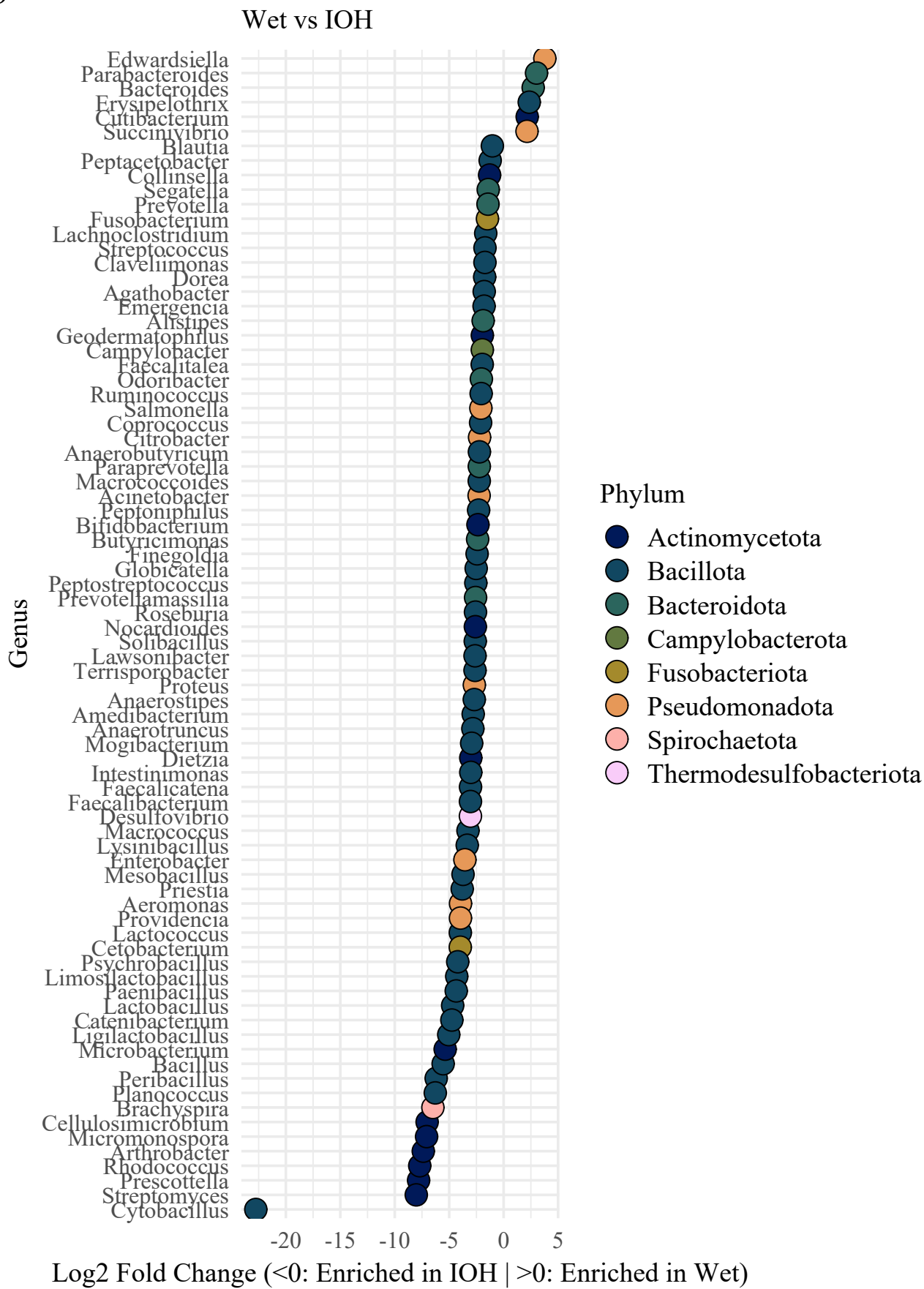

Supplement: Supplementary file 2 — Supplementary Material 2: Additional file 2. Host-stratified DESeq2 differential abundance results for lions (PDF). Genus-level differential abundance analysis comparing Wet vs. IOS (panel a) and Wet vs. IOH (panel b) samples in lions. Points represent significantly differentially abundant genera (adjusted p < 0.05), colored by phylum. Log2 fold change values indicate enrichment relative to Wet samples (negative values indicate enrichment in IOS or IOH; positive values indicate enrichment in Wet). [file 42523_2026_553_MOESM2_ESM.pdf]

# DESeq2 differential abundance by sample condition (Spotted\_hyena)

a

Wet vs IOS

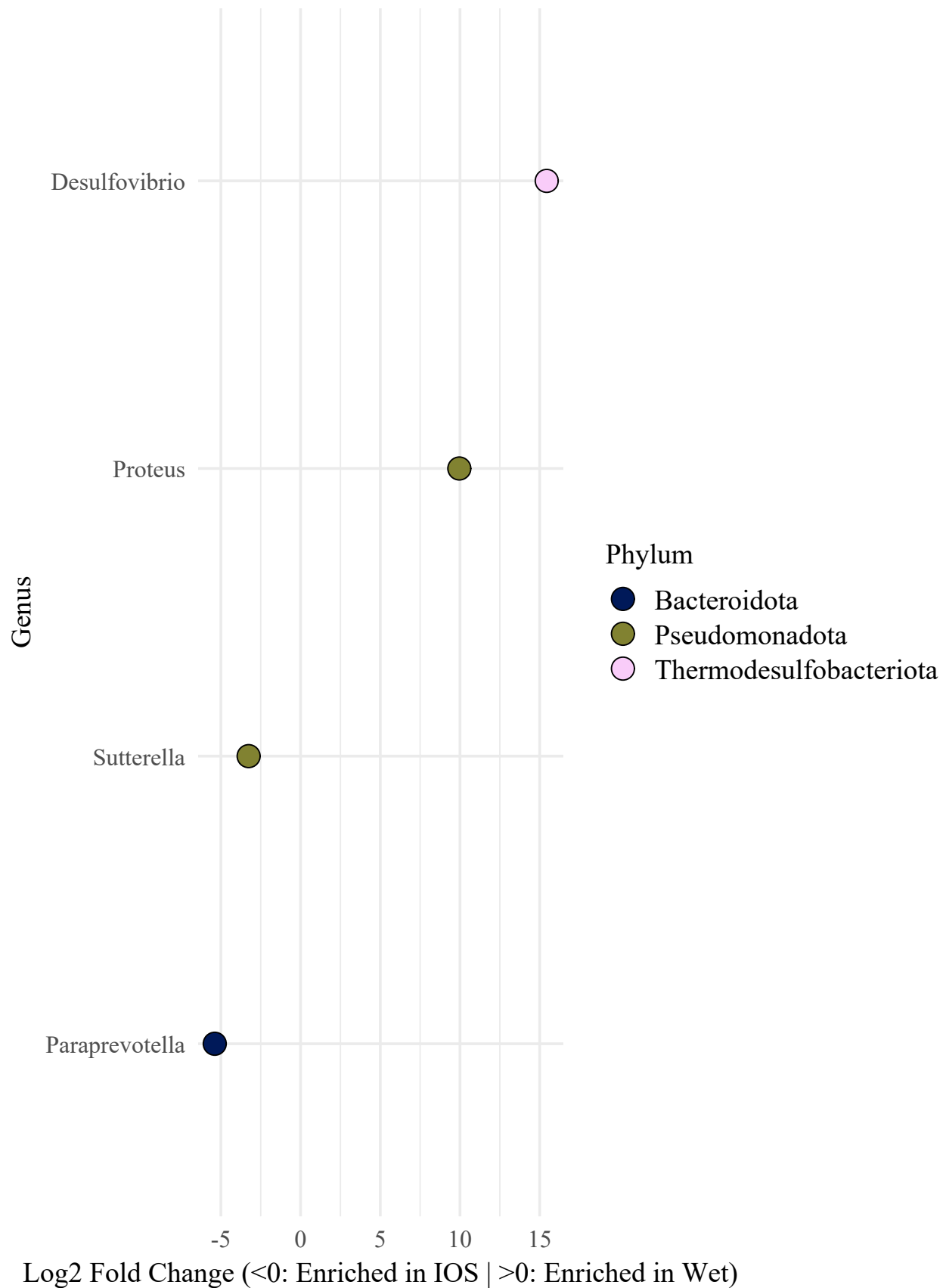

b

Wet vs IOH

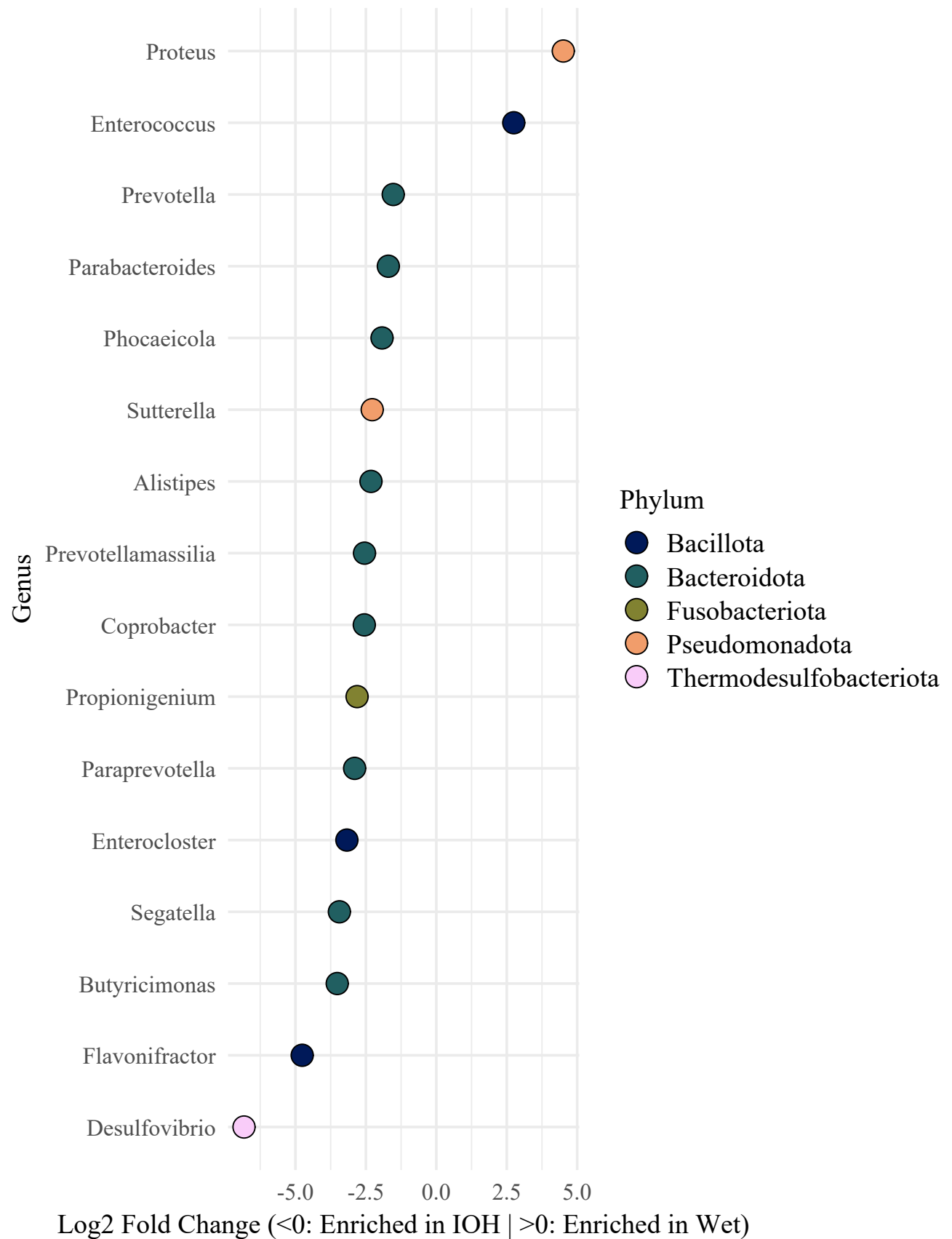

Supplement: Supplementary file 3 — Supplementary Material 3: Additional file 3. Host-stratified DESeq2 differential abundance results for spotted hyenas (PDF). Genus-level differential abundance analysis comparing Wet vs. IOS (panel a) and Wet vs. IOH (panel b) samples in spotted hyenas. Points represent significantly differentially abundant genera (adjusted p < 0.05), colored by phylum. Log2 fold change values indicate enrichment relative to Wet samples (negative values indicate enrichment in IOS or IOH; positive values indicate enrichment in Wet). [file 42523_2026_553_MOESM3_ESM.pdf]

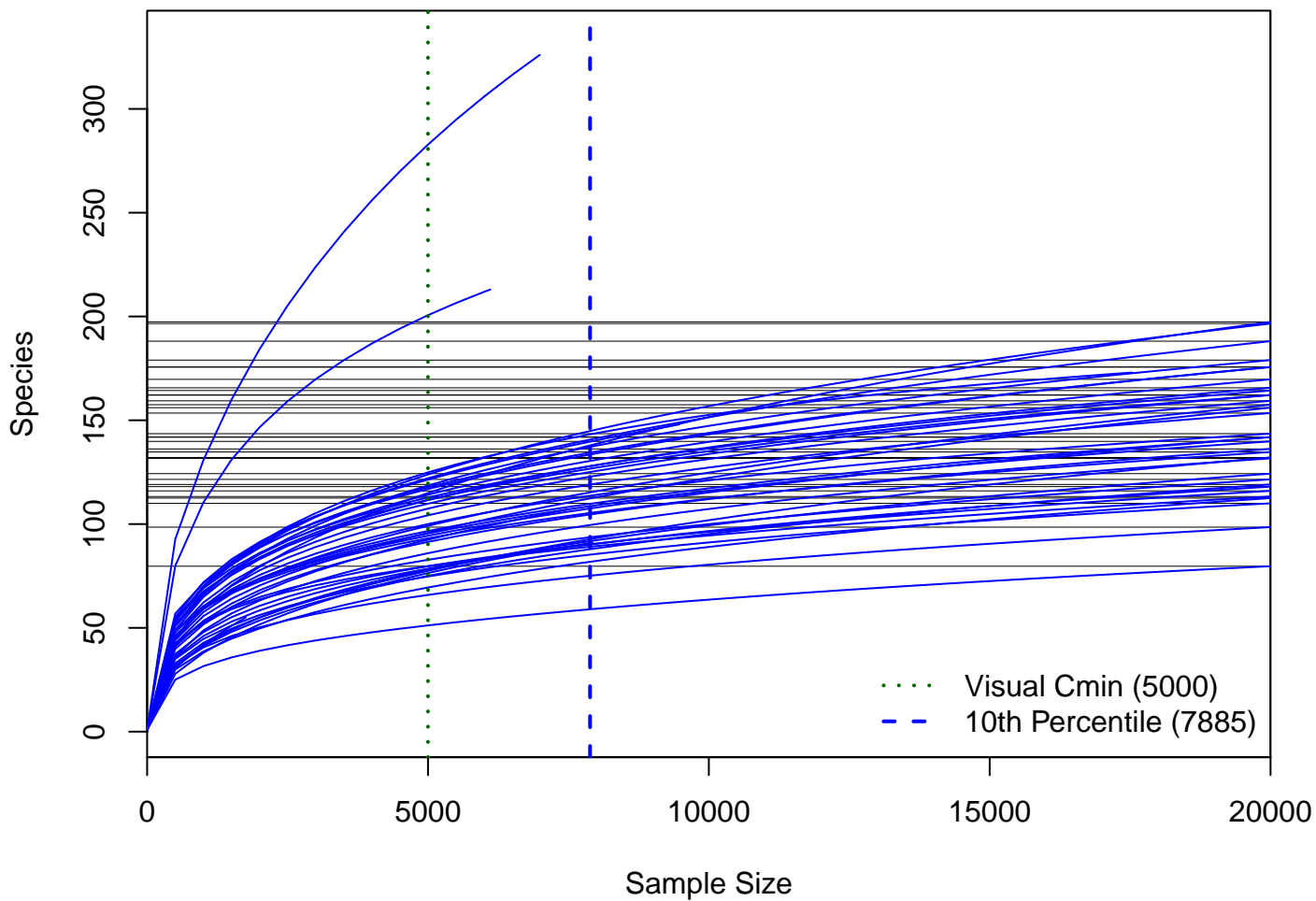

Supplement: Supplementary file 4 — Supplementary Material 4: Additional file 4. Rarefaction curves for storage technique comparison (PDF). Rarefaction curves showing sequencing depth for all samples included in the storage technique analysis. Vertical dashed lines indicate the selected rarefaction threshold (5,000 reads) and the 10th percentile sequencing depth (7,885 reads). [file 42523_2026_553_MOESM4_ESM.pdf]

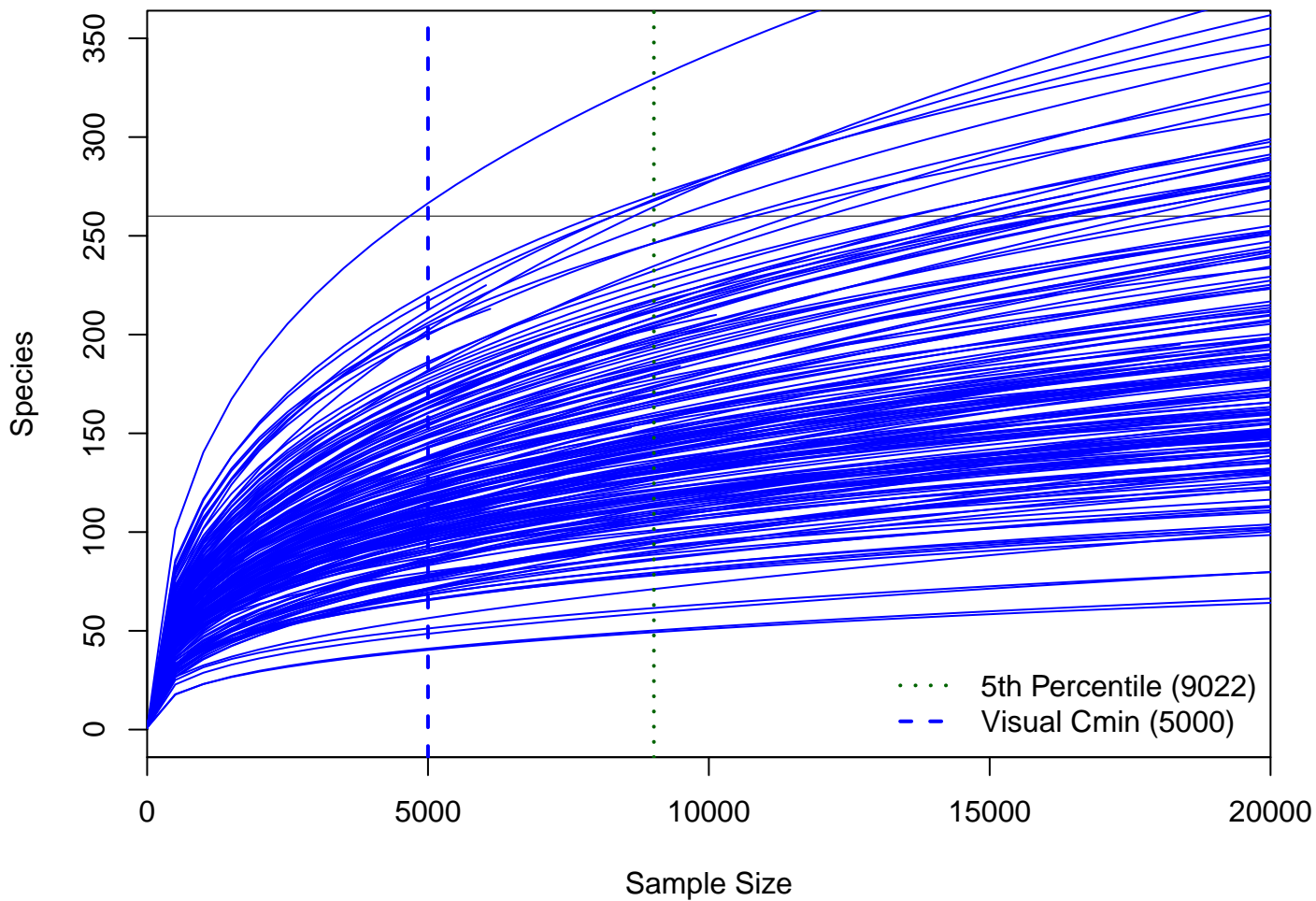

Supplement: Supplementary file 5 — Supplementary Material 5: Additional file 5. Rarefaction curves for sample condition comparison (PDF). Rarefaction curves showing sequencing depth for samples included in the sample condition analysis. Vertical dashed lines indicate the selected rarefaction threshold (5,000 reads) and the 5th percentile sequencing depth (9,022 reads). [file 42523_2026_553_MOESM5_ESM.pdf]
